# Supplementary material for: Giant chiral magnetoelectric oscillations in a van der Waals multiferroic
Source: Nature. 2024 Jul 17;632(8024):273–9. doi: 10.1038/s41586-024-07678-5 (PMC11306099; doi:10.1038/s41586-024-07678-5)
Supplement: Supplementary file 1 — This file contains Supplementary Notes 1–7, Supplementary Figs. 1–20, Supplementary Tables 1–7 and Supplementary References. [file 41586_2024_7678_MOESM1_ESM.pdf]

nature portfolio

https://doi.org/10.1038/s41586-024-07678-5

Supplementary information

Giant chiral magnetoelectric oscillations in a van der Waals multiferroic

In the format provided by the authors and unedited

Supplementary Information for: "Giant chiral magnetoelectric oscillations in a van der Waals multiferroic"

Frank Y. Gao[1,†], Xinyue Peng[1,†], Xinle Cheng[2], Emil Viñas Boström[2,3], Dong Seob Kim[1], Ravish K. Jain[4], Deepak Vishnu[4,5], Kalaivanan Raju[4], Raman Sankar[4], Shang-Fan Lee[4], Michael A. Sentef[2,6], Takashi Kurumaji[7], Xiaoqin Li[1], Peizhe Tang[2], Angel Rubio[2,3,8,*], Edoardo Baldini[1,*]

[1]*Department of Physics and Center for Complex Quantum Systems, The University of Texas at Austin, Austin, TX, USA*

[2]*Max Planck Institute for the Structure and Dynamics of Matter, Hamburg, Germany*

[3]*Nano-Bio Spectroscopy Group, Departamento de Física de Materiales, Universidad del País Vasco, San Sebastián, Spain*

[4]*Institute of Physics, Academia Sinica, Taipei, Taiwan*

[5]*Department of Chemistry, National Tsing Hua University, Hsinchu, Taiwan*

[6]*Institute for Theoretical Physics and Bremen Center for Computational Materials Science, University of Bremen, Bremen, Germany*

[7]*Division of Physics, Mathematics and Astronomy, California Institute of Technology, Pasadena, CA, USA*

[8]*Center for Computational Quantum Physics, The Flatiron Institute, New York, NY, USA*

[†]*These authors contributed equally to this work.*

[*]*Corresponding authors:*

edoardo.baldini@austin.utexas.edu, angel.rubio@mpsd.mpg.de

June 21, 2024

Contents

Supplementary Note 1: Sample characterization 3

Supplementary Note 2: Details on magnon and phonon calculations 7

Supplementary Note 3: Microscopic source terms for SHG 16

Supplementary Note 4: Modelling of SHG Data 18

Supplementary Note 5: Comparison between tr-RKerr and tr-SHG signals 25

Supplementary Note 6: Dynamical magnetoelectric coupling 33

Supplementary Note 7: Pump polarization dependence 37

Supplementary Figures 38

References 42

Supplementary Note 1: Sample characterization

Structural and magnetic characterization of bulk NiI_2. We studied the crystal structure of bulk NiI_2 single crystals using X-ray diffraction (XRD). Figure S1a shows the XRD pattern collected along the (001) plane, which is consistent with previously reported XRD measurements of NiI_2 single crystals[1,2]. We also investigated the magnetic behavior of the crystals using a superconducting quantum interference device (SQUID) magnetometer (Quantum Design Inc.) with the applied magnetic field along the out-of-plane crystallographic direction. Figure S1b shows the variation of the inverse molar magnetic susceptibility ($1/\chi_m$) (blue curve) and of the quantity $\chi_m T$ (red curve) with temperature T. The two kinks in the $1/\chi_m$ response at 75 K and 60 K correspond to distinct phase transitions. We ascribe the first kink to the transition from a paramagnetic to a collinear antiferromagnetic phase, and we relate the second kink to the transition from the collinear antiferromagnetic to the spin-spiral phase. A straight line fit (blue dashed line in Fig. S1b) of the $1/\chi_m$ versus T data in the high-temperature region (280-300 K) yields a positive T-intercept (64.26 K) which suggests ferromagnetic nearest-neighbor coupling. As the temperature is lowered, the T-intercept for the linear fit of the data moves toward negative values, and in the low-temperature region (10-20 K) it becomes negative (-92.97 K). The latter trend is consistent with the helimagnetic arrangement reported in the literature[2,3]. Figure S1c displays the magnetic field dependence of the magnetization at 10 K and 300 K, respectively. An S-like-shaped curve lacking hysteresis is observed, and the change in slope around zero field is more pronounced in the room temperature curve.

Surface quality of exfoliated NiI_2 sample. To establish the surface quality of our NiI_2 flakes, we performed atomic force microscopy (AFM) in a glovebox filled with nitrogen. The optical and AFM images of a representative flake are shown in Fig. S2a,b. Examination of the highlighted white lines 1 and 2 in Fig. S2b reveals a clean step profile and a uniform surface with a roughness of 0.30 nm, respectively. Such profiles are displayed in Fig. S2c and Fig. S2d.

Sample stability upon photoexcitation. To ensure that we probed the same enantiopure domain during each pump-probe scan and to exclude any potential dephasing of the underlying domain, we characterized the static optical second harmonic generation (SHG) polarimetry signals before and after each time-resolved measurement. We found that the static SHG signal remained essentially unchanged over time, even after three days of exposure to optical pump and probe pulses (Fig. S3). Additionally, as evidence of the sample's stability under our photoexcitation conditions, we monitored the time-resolved SHG (tr-SHG) signal scan-by-scan. Upon comparing these scans, we observed that the polarized tr-SHG signals were stable even after 18 hours of measurements in the same polar configuration (Fig. S4).

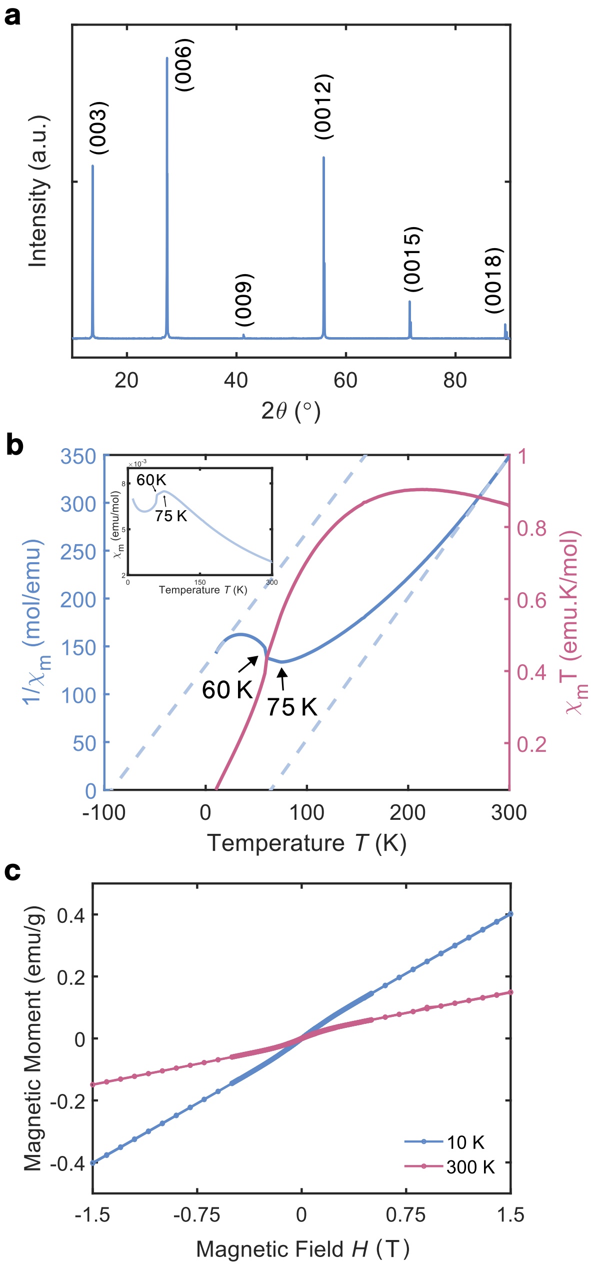

Fig. S1 | Structural and magnetic characterization of bulk NiI_2. a, Single-crystal XRD of NiI_2 along the (001) plane. **b,** Variation of $1/\chi_m$ and $\chi_m T$ with temperature T at an applied out-of-plane magnetic field of 7 T. The inset is the χ_m versus T graph. The blue dashed lines are linear fits of the $1/\chi_m$ versus T data in the high-temperature (280-300 K) and low-temperature (10-20 K) regions. **c,** M-H curves of the NiI_2 bulk crystal with a magnetic field ranging from -1.5 T to 1.5 T applied along the out-of-plane axis at 10 K (*blue*) and 300 K (*red*).

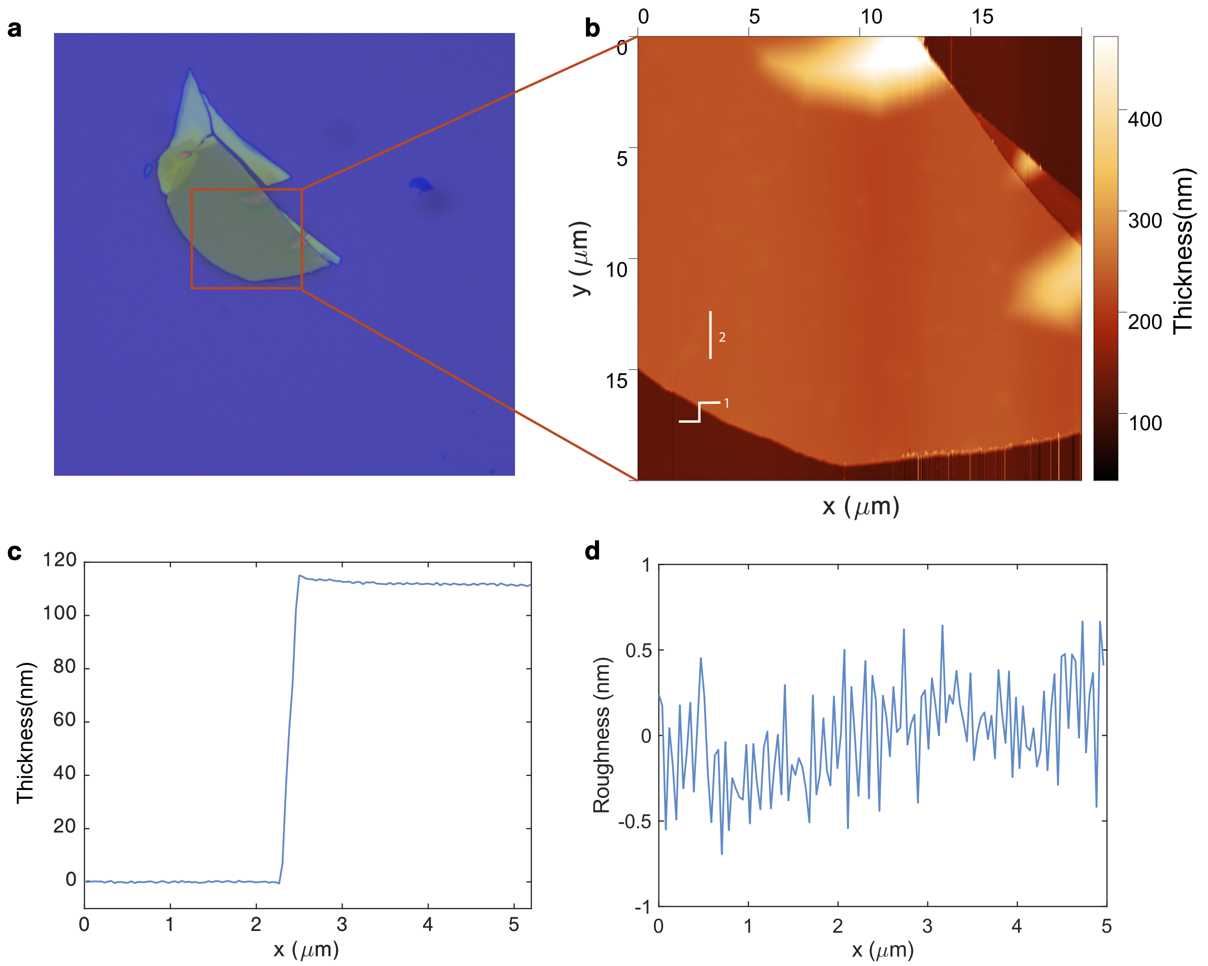

Fig. S2 | Surface quality examination. a, Optical microscopy and **b,** AFM images of a NiI_2 flake. Line-cut profiles along the white lines **c,** 1 and **d,** 2 as indicated in panel **b**.

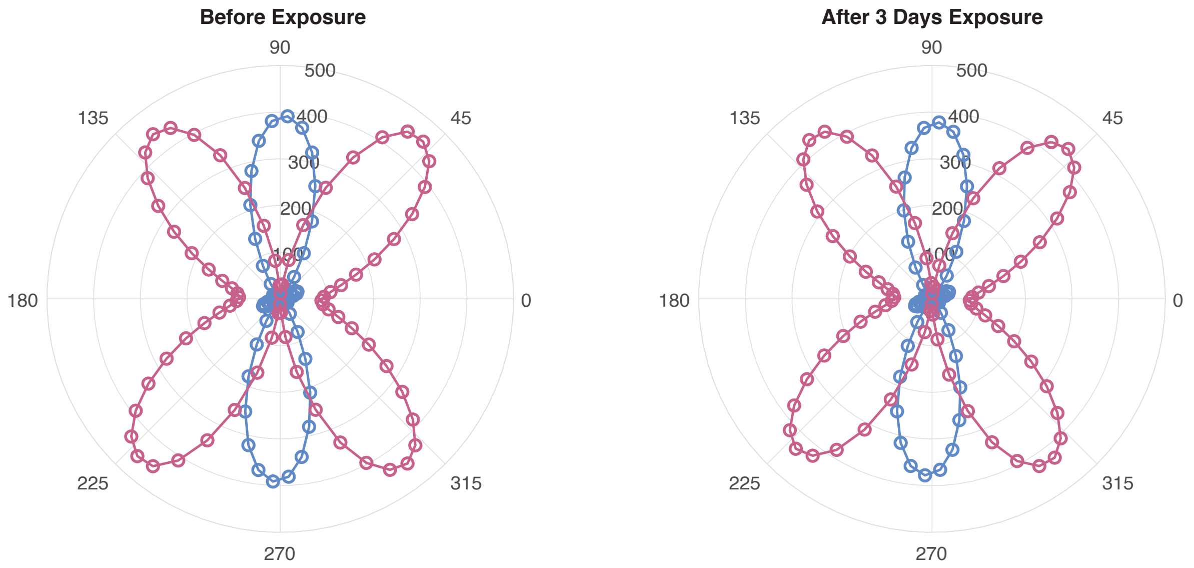

Fig. S3 | Static SHG signal stability. Static SHG polarimetry signal taken at 2.4 K on an enantiopure domain of NiI_2 before (*left*) and after (*right*) exposing the domain to $\sim$3 days of irradiation with the 1.13 eV pump beam (used for tr-SHG measurements) and the 1.20 eV probe beam.

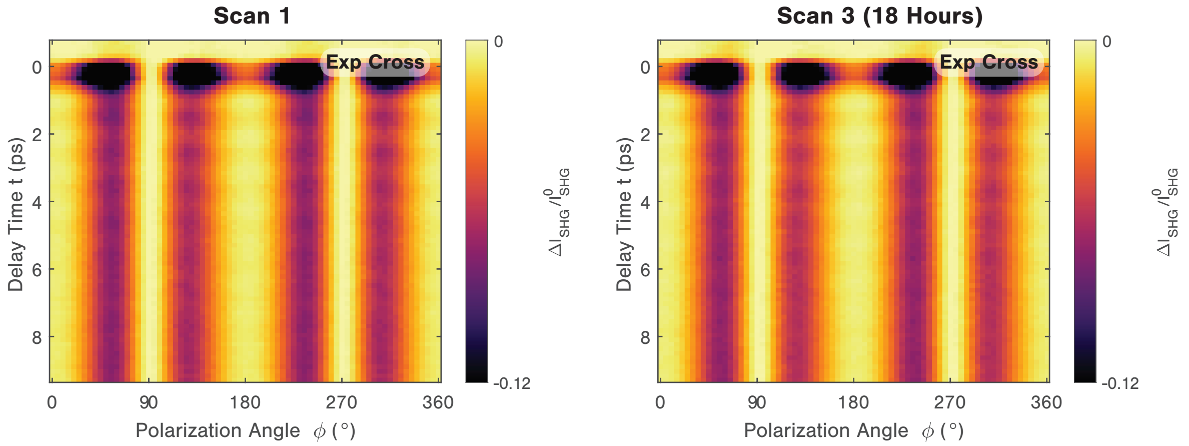

Fig. S4 | Tr-SHG polarimetry signal stability. Individual raw anisotropic tr-SHG scans showing the signals collected in the cross-polarized configurations at the beginning of the acquisition (*left*) and 18 hours later (*right*). Data in Fig. 2d,f of the main text displays the oscillatory component extracted from the average of all such raw scans. These transient signals are normalized to the maximum static SHG signal of $\sim$ 500 μV.

Supplementary Note 2: Details on magnon and phonon calculations

Spin Hamiltonian. We studied the magnetic properties of NiI_2 by neglecting interlayer interactions. The resulting spin Hamiltonian describing a single magnetic layer is[4]

$$H_S = H_S^{\mathrm{iso}} + H_S^{\mathrm{ani}}, \tag{1}$$

where H_S^{iso} is the dominant isotropic part that can be written as

$$H_S^{\mathrm{iso}} = \frac{1}{2}\sum_{ij} J_{ij}\mathbf{S}_i \cdot \mathbf{S}_j + \frac{B}{2}\sum_{\langle ij \rangle}(\mathbf{S}_i \cdot \mathbf{S}_j)^2. \tag{2}$$

Here, $\mathbf{S}_i$ is the spin of a Ni ion at position $\mathbf{r}_i$, and J_{ij} are isotropic exchange interactions extending up to the third nearest neighbors (denoted by J_1, J_2, and J_3). The competition between the ferromagnetic nearest-neighbor exchange interaction and the antiferromagnetic third nearest-neighbor exchange results in a spin-spiral ground state. The parameter $B < 0$ determines the nearest-neighbor ferromagnetic biquadratic exchange coupling, favoring collinear order.

In addition, Eq. 1 contains the sub-dominant anisotropic Hamiltonian

$$H_S^{\mathrm{ani}} = \frac{1}{2}\sum_{ij} \mathbf{S}_i \cdot (\mathbf{J}_{ij}^r \mathbf{S}_j) + \frac{1}{2}\sum_{i} \mathbf{S}_i \cdot (\mathbf{A}_s \mathbf{S}_i), \tag{3}$$

where $\mathbf{J}_{ij}^r$ is a traceless and symmetric matrix describing nearest ($\mathbf{J}_1^r$) and third nearest ($\mathbf{J}_3^r$) neighbor anisotropic exchange, and $\mathbf{A}_s$ is a matrix quantifying the single-ion anisotropy. H_S^{ani} breaks the rotational symmetry of the spins and fixes the global orientation of the ground state. We note that the Hamiltonian here is written in the cubic Cartesian axes. Another common convention for triangular lattices is to write the model in local crystallographic axes (see *e.g.*, Ref. [5] for a discussion of the relation between these bases). In the local picture, the anisotropic Hamiltonian generates a Kitaev interaction K, as well as the so-called Γ and Γ' interactions. In our work, we find that operating in the cubic basis set is more convenient. All the parameters stated below refer to this basis.

We obtained these exchange parameters from first principles using density functional theory (DFT), as described in the Methods section of the main text. The values of the exchange interactions and the single-ion anisotropy are given in Table S1. The values are consistent with those previously reported [4] (see discussion below) and reproduce the spin-spiral ground state observed experimentally.

Magnon modes. We obtained the low-energy spin-wave excitations by solving the Landau-Lifshitz equation

$$\frac{\partial \mathbf{S}_i}{\partial t} = -\mathbf{S}_i \times \frac{\delta H_S}{\delta \mathbf{S}_i}, \tag{4}$$

where H_S is the spin Hamiltonian introduced above. As elaborated in the main text, two spin waves at the Brillouin zone center exhibit opposite parity under the C_2 symmetry transformation. The C_2-odd magnon (EM_o) is found to have an energy of 3.93 meV, while the C_2-even

J_1	J_2	J_3	B
-5.03	-0.32	3.95	-0.89
$J^r_{1,xx}$	$J^r_{1,yy}$	$J^r_{1,zz}$	$J^r_{1,xy}$
-0.13	0.82	-0.69	-0.96
$J^r_{3,xx}$	$J^r_{3,yy}$	$J^r_{3,zz}$	$J^r_{3,xy}$
0.08	-0.02	-0.06	-0.045
A_{zz}			
0.44			

Table S1 | Parameters of the spin Hamiltonian. Symmetry-allowed magnetic exchange parameters and single-ion anisotropy calculated from first principles. The spin axes are aligned such that the y-axis is along a Ni-Ni bond and the z-axis is the out-of-plane direction. The energy unit is meV. As the second nearest-neighbor exchange is small, only its average value over all components is shown.

magnon (EM$_e$) lies at an energy of 4.30 meV. We assign these two magnon modes to the collective excitations observed in our measurements. To support this statement, we first rule out the possibility of hybridized phonon modes and then use both DFT calculations and a model derived from an effective spin cluster to show that these magnon modes acquire large electric dipole moments through the inverse Dzyaloshinskii–Moriya interaction (DMI) mechanism (spin-current mechanism)[6].

Phonon modes and magnon-phonon coupling. We performed DFT calculations to obtain the phonon dispersion of monolayer NiI_2 in a ferromagnetic configuration. As illustrated in Fig. S5, the optical phonons at the Brillouin zone center have energies larger than 8 meV, and therefore lie above the energies of the two modes detected experimentally. While our calculations were conducted in the monolayer structure and did not account for interlayer shear modes, it is important to note that these modes are expected to appear at much lower energies in bulk NiI_2[7]. Furthermore, interlayer phonons would be present around a similar energy at any temperature[7], unlike the electromagnon modes observed in our measurements.

We also ruled out the possibility that zone-folded acoustic phonons hybridize with the magnon modes. In a spiral magnet, the folded acoustic phonons are forbidden to modulate any translationally invariant observables by the generalized Bloch theorem[8], which is only weakly broken by spin-orbit coupling. This statement is also supported experimentally, as we did not observe any sign of folded acoustic phonons in the Raman spectrum, indicating a vanishing Raman tensor.

To numerically verify the lack of strong magnon-phonon interactions, we directly extracted the coupling strength between magnon and phonon modes using the approach described in Ref. [9]. We defined the hybridization strength as $h_{mp} = X_{mp}/|\epsilon_m - \epsilon_p|$, where X_{mp} is the magnon-phonon coupling strength, and $|\epsilon_m - \epsilon_p|$ is their energy difference. We obtained a value of $h_{mp} < 0.2$ between the two low-energy magnons and all phonon modes. Such small hybridization can be attributed to the large radius of the I atom and the relatively small radius of the Ni d-shell. Furthermore, a frozen-phonon calculation revealed that folded acoustic phonons generally have an electric dipole one order of magnitude smaller than the electro-

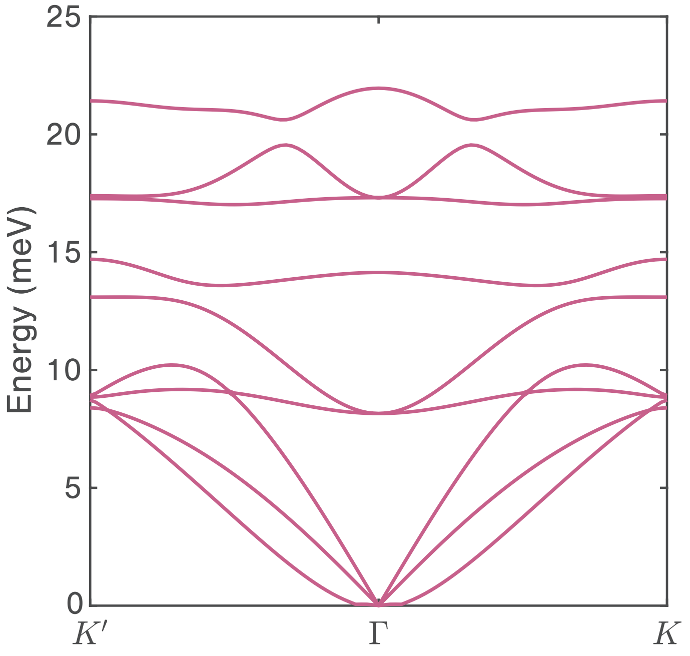

Fig. S5 | Calculated phonon dispersion. Energy-momentum dispersion relation of the phonon modes of monolayer NiI_2 obtained from DFT calculations in a ferromagnetic configuration.

magnon modes. This further rules out the role of phonons in the dynamical magnetoelectric response.

Electric polarization of the (electro-)magnon modes. We also demonstrated that the low-energy magnon modes are associated with a finite electric polarization and are, therefore, electromagnons. We achieved this through two different approaches: (i) by performing first-principles calculations of the magnon electric dipole moments, and (ii) by an analytical generalization of the spin current model presented in Ref. [6], adapted to the present context. The latter approach shows that the electric polarization emerges as a consequence of the non-collinear spin order and spin-orbit interactions, in agreement with recent proposals [10].

To obtain the electric dipoles associated with the magnon modes, we first calculated the static polarization of monolayer NiI_2 via the modern theory of polarization[11]. We found that the electric polarization has a magnitude of $P_{\text{el}} = 5.3 \times 10^{-13}$ C/m and is directed along the [010] direction. For a three-dimensional unit cell, this polarization corresponds to a value of approximately 8.0×10^{-4} C/m^2. This is consistent with our symmetry analysis (see below) and with previous works[10].

We then performed restricted DFT calculations, where the directions of the spins were modulated according to the patterns of the magnon modes EM_o and EM_e. This treatment allowed us to compute the change in electric polarization induced by the magnons. Within such a frozen-magnon approximation, we could associate an effective electric dipole moment with each magnon mode. We found that mode EM_o has an electric dipole moment of magnitude $d_o = 2.5\ \mu_B/c$ perpendicular to the static electric polarization, while mode EM_e has an electric

dipole moment of magnitude $d_e = 10.3\ \mu_B/c$ parallel to the static electric polarization. Here, μ_B is the Bohr magneton and c is the speed of light. These results are again consistent with the C_2-symmetry analysis and show that the two low-energy magnon modes are indeed electromagnons with colossal electric dipole moments that couple strongly to an incident electric field.

To understand the origin of the electromagnons' electric polarization, we derived an analytical generalization of the spin-current model introduced by Katsura *et al.* [6] and later expanded to helical triangular antiferromagnets[12], adapted to NiI_2. The main differences between the previous theory and our model are that the spin-orbit interaction in NiI_2 acts on the ligand (instead of the transition metal ion) and that the transition metal-ligand bonds are at approximately $90°$ (instead of $180°$). In line with Ref. [6], we considered an atomic cluster consisting of two Ni atoms and one I atom (as indicated in Fig. S6). Working in the hole picture, the e_g orbitals on each Ni atom are occupied by two holes each, while the six p-orbitals on each I atom are empty. Due to the orbital symmetry, we only had to consider the p-orbitals on the I atoms that lie in the Ni-I-Ni plane (labeled according to the axes shown in Fig. S6), and the two e_g orbitals of Ni that lie along the Ni-I bonds. Moreover, we treated the Hubbard interaction and Hund's coupling on the Ni atoms within the mean-field approximation, which is equivalent to imposing a large staggering field along the direction of the spin vector $\mathbf{S}_i$ on each Ni. Therefore, the low-energy subspace is spanned by the six orbitals $\{|d_1\rangle, |d_2\rangle, |p_{1\uparrow}\rangle, |p_{1\downarrow}\rangle, |p_{2\uparrow}\rangle, |p_{2\downarrow}\rangle\}$, where $|d_1\rangle$ and $|d_2\rangle$ are the d-orbitals polarized along $\mathbf{S}_1$ and $\mathbf{S}_2$. In addition, $|p_{1\uparrow}\rangle, |p_{1\downarrow}\rangle$ and $|p_{2\uparrow}\rangle, |p_{2\downarrow}\rangle$ are p-orbitals directed along $\hat{\mathrm{e}}_1$ and $\hat{\mathrm{e}}_2$, respectively.

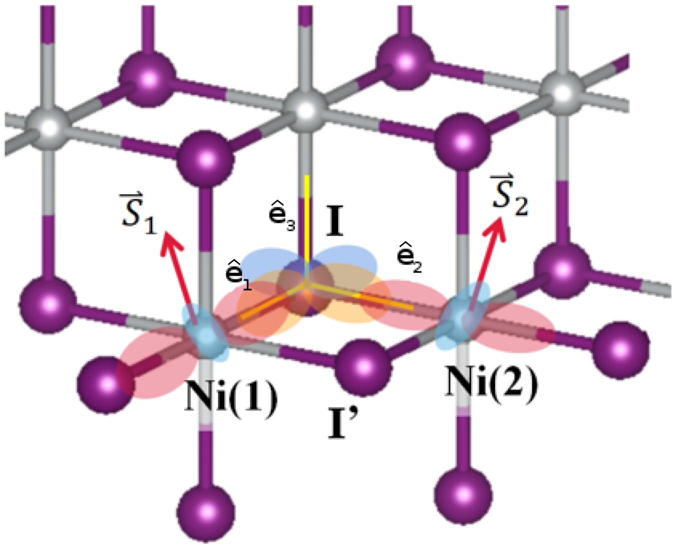

Fig. S6 | Illustration of the Ni-I-Ni atomic cluster considered in our calculations. The shaded areas indicate the relevant atomic orbitals. $\mathbf{S}_1$ and $\mathbf{S}_2$ denote the spins on the two neighboring Ni atoms. The local $\hat{\mathrm{e}}_1$, $\hat{\mathrm{e}}_2$, and $\hat{\mathrm{e}}_3$ axes are also shown.

Under the above assumptions, the Hamiltonian of the Ni-I-Ni cluster consists of three parts: $H_{\text{cluster}} = H_{\text{a}} + H_{\text{d-p}} + H_{\text{so}}$, where H_{a} describes the isolated atoms, $H_{\text{d-p}}$ the d-p hybridization, and H_{so} the spin-orbit coupling on I (which is dominant due to the large atomic

number of I compared to Ni). The different contributions to the Hamiltonian are

$$\begin{aligned} H_{\mathrm{a}} &= -\Delta(d_1^\dagger d_1 + d_2^\dagger d_2) \\ H_{\mathrm{d-p}} &= t\sum_\sigma \left[\chi_{1\sigma} p_{1\sigma}^\dagger d_1 + \chi_{2\sigma} p_{2\sigma}^\dagger d_2 + H.c.\right] \\ H_{\mathrm{so}} &= i\lambda(p_{1\downarrow}^\dagger p_{2\downarrow} - p_{1\uparrow}^\dagger p_{2\uparrow}) + H.c., \end{aligned} \tag{5}$$

where Δ is the charge-transfer energy, t the d-p hopping amplitude, and λ the strength of the spin-orbit coupling. The two-component spinors $\chi_{i\sigma}$ describe the components of the Ni spin $\mathbf{S}_i$. The operator for the electric polarization is

$$\begin{aligned} \hat{\mathbf{P}} = \mathcal{I}_1 &\sum_\sigma \left[\chi_{1\sigma} p_{2\sigma}^\dagger d_1 \hat{\mathbf{e}}_2 + \chi_{2\sigma} p_{1\sigma}^\dagger d_2 \hat{\mathbf{e}}_1 + H.c.\right] \\ + \mathcal{I}_2 &\sum_\sigma \left[\chi_{1\sigma} p_{1\sigma}^\dagger d_1 \hat{\mathbf{e}}_1 + \chi_{2\sigma} p_{2\sigma}^\dagger d_2 \hat{\mathbf{e}}_2 + H.c.\right], \end{aligned} \tag{6}$$

where $\hat{\mathbf{e}}_1$ and $\hat{\mathbf{e}}_2$ are unit vectors along the Ni-I bonds shown in Fig. S6, and $\mathcal{I}_1$ and $\mathcal{I}_2$ are overlap integrals defined by

$$\begin{aligned} \mathcal{I}_1 &= e\int d\mathbf{r}(\mathbf{r}\cdot\hat{\mathbf{e}}_2)p_2^*(\mathbf{r})d_1(\mathbf{r}) \\ \mathcal{I}_2 &= e\int d\mathbf{r}(\mathbf{r}\cdot\hat{\mathbf{e}}_1)p_1^*(\mathbf{r})d_1(\mathbf{r}). \end{aligned} \tag{7}$$

Considering the spin-orbit Hamiltonian H_{so} as a perturbation to the remaining terms $H_0 = H_{\mathrm{a}} + H_{\mathrm{d-p}}$, the change in polarization due to the magnetic order is given to leading order by

$$\hat{\mathbf{P}}^{(1)} = \frac{\lambda t^3}{\Delta^4}\mathcal{I}_1(\hat{\mathbf{e}}_1 + \hat{\mathbf{e}}_2)[\hat{\mathbf{e}}_3\cdot(\mathbf{S}_1\times\mathbf{S}_2)]. \tag{8}$$

Summing over all the bonds of the magnetic unit cell yields a total polarization along the $[010]$ axis, in agreement with our first-principles calculations. From Eq. 8, we note that the polarization tends to zero for vanishing spin-orbit interaction or collinear spins. Therefore, both spin-orbit interactions and a non-collinear magnetic order are necessary ingredients to induce a macroscopic polarization in NiI_2. This expression also provides the electric polarization associated with each magnon mode, which agrees with our first-principles calculations and again confirms that these modes are indeed electromagnons.

Using Eq. 8, we calculated the time-resolved modulation of electric polarization by the two electromagnon modes and compare it with the transient magnetization of these two modes, as illustrated in Fig. S7. The C_2 parity enforces constraints on the components of electric polarization and magnetization that the two EM modes modulate. In the following, we define the axes x- y- and z-axis in line with the convention used in the main text (see Fig. 1c). Namely, we take the x-axis to lie along the in-plane spiral wave vector, the y-axis to be along the electric polarization and the z-axis to be along the out-of-plane direction. Thus, the y-axis is parallel to the C_2-axis, and the C_2-even mode (EM_e) only modulates the amplitude of the y-component of electric polarization and magnetization (Fig. S7a), while the C_2-odd mode (EM_o) modulates the amplitude of the x- and z-components of the electric polarization and magnetization (Fig. S7b).

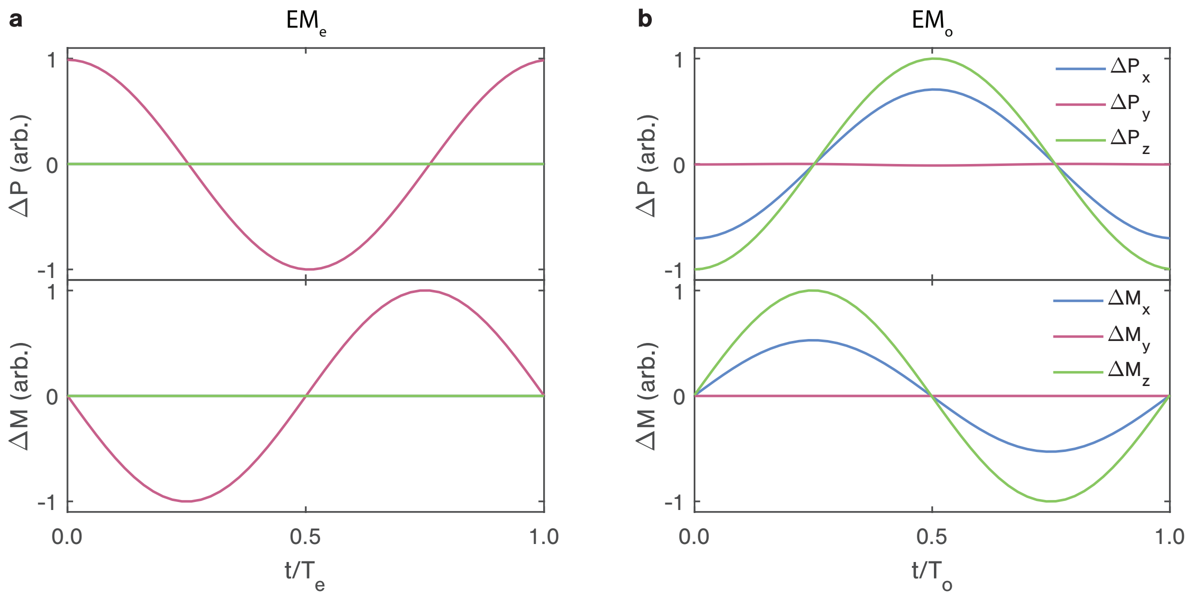

Fig. S7 | Electromagnon dynamics. Calculated polarization dynamics (ΔP) and magnetization dynamics (ΔM) for **a,** EM_e and **b,** EM_o, as obtained from DFT calculations using the frozen-magnon approximation. T_e and T_o represent the oscillation periods of EM_e and EM_o, respectively.

Another prominent feature from Fig. S7 is that the modulation of electric polarization and magnetization differs by a phase of $\pm\pi/2$. This $\pi/2$ phase arises from the quasi-time-reversal symmetry $\tilde{T}$ of the system. $\tilde{T}$ consists of ordinary time-reversal combined with a half-spiral translation along the vector **q**, *i.e.*, parallel to the spiral wave vector but shifted by half the spiral period. The electric polarization and magnetization transform under $\tilde{T}$ as even and odd, respectively; this implies that their complex amplitudes necessarily differ by a factor of i, leading to the $\pi/2$ phase shift.

Dependence of the magnetoelectric properties on the spin-plane canting angle and magnetic parameters. To demonstrate the robustness of our results to variations in the magnetic parameters, in Tab. S2 we compare the spin parameters calculated in this work with the values recently reported in Ref. [13]. In our work, we used the Perdew-Burke-Erzenhof (PBE) functional together with the four-state method[14–16] to extract the spin parameters, while the work of Ref. [13] considers either the PBE or hybrid HSE06 (HSE) functional together with a machine learning approach. Interestingly, our values are closer to those of Ref. [13] obtained with the HSE functional rather than the PBE functional, indicating a dependence of the parameters on both the functional and the method used to extract them. However, since all parameter sets are in close agreement, both the electromagnon energies and electric polarizations are expected to be insensitive to the choice of functional and calculation method.

To support this statement, we note that the most sensitive property in our model calculations is the canting angle of the spin rotation plane with respect to the monolayer plane. Indeed, Ref. [13] found that this angle depends sensitively on the ratio between the nearest-neighbor and third nearest-neighbor exchanges J_1 and J_3, with a larger value of J_3/J_1 favoring a larger canting angle. We observe that our model predicts an angle of $45°$ compared to the experimentally measured angle of $55°$. However, to compensate for this effect, we performed our DFT calcu-

	J_1	J_2	J_3	K	B
Ref. [13] (PBE, ML)	-4.338	-0.121	3.155	1.433	-0.685
Ref. [13] (HSE, ML)	-4.976	-0.155	2.250	0.858	-0.719
This work (PBE, 4S)	-5.030	-0.320	3.950	0.960	-0.890

Table S2 | Comparison of spin parameters calculated with different exchange-correlation potentials and computational schemes. The functionals are either the generalized gradient approximation (GGA) potential of Perdew, Burke and Erzenhof (PBE) or the hybrid functional HSE06. The computational approach to extract the spin parameters are either a fit using machine learning (ML) or the four-state method (4S). J_i denote isotropic exchange interactions between ith nearest neighbors, K is the nearest neighbor Kitaev interaction, and B is the nearest neighbor biquadratic exchange. The energy unit is meV.

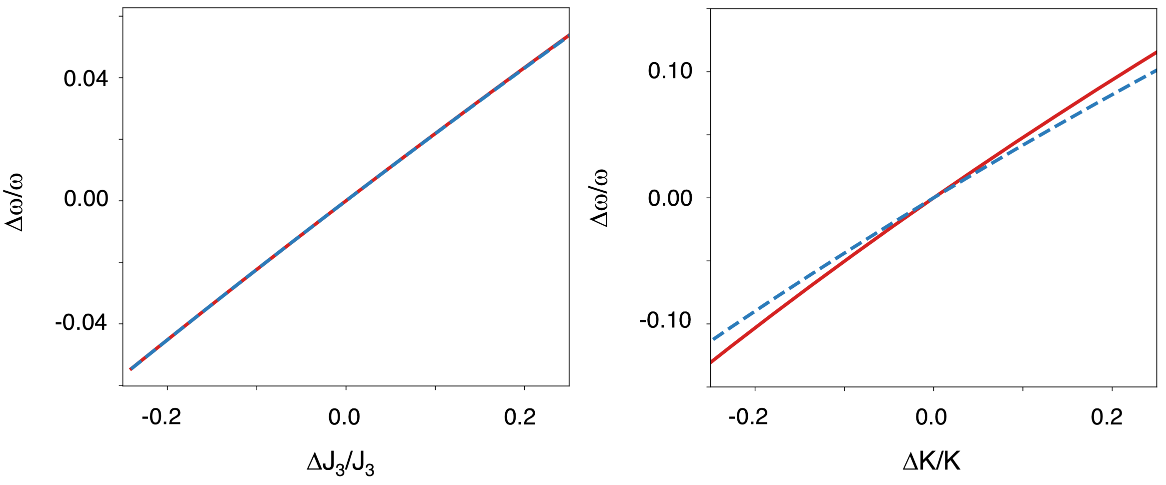

Fig. S8 | Dependence of the electromagnon energies on the third nearest-neighbor exchange parameter J_3 and the nearest-neighbor Kitaev interaction K. The electromagnon energies have an approximately linear scaling on the parameters: specifically, the energies of electromagnons increase by ~10% when J_3 and K increase by ~20%. The blue and red curves correspond to EM_o and EM_e, respectively.

lations in the experimental magnetic configuration and still found a similar value of the electric polarization within our model (with $P_{\mathrm{DFT}} = 5.3 \times 10^{-13}$ C/m and $P_{\mathrm{model}} = 4.1 \times 10^{-13}$ C/m). Furthermore, from Eq. 8, we see that the electric polarization changes with the canting angle as $P = P_0 \sin\theta$, where θ is the angle between the spiral propagation vector $\mathbf{q}$ and its projection $\mathbf{q}_{\parallel}$ onto the monolayer plane. This demonstrates that the change in polarization with respect to θ is approximately linear, such that small changes in θ lead to small changes in P. In addition, this result indicates that our theory slightly underestimates the electric polarization, since $\sin(45°) < \sin(55°)$.

A similar argument can be made for the electromagnon energies. We note that the parameter set used in our work has slightly larger values of the nearest-neighbor and third nearest-neighbor exchanges J_1 and J_3, compared to the model in Ref. [13]. In Fig. S8, we show the change in electromagnon energies when varying the third-nearest neighbor parameter J_3 and the Kitaev interaction K, keeping J_1 fixed to define the overall unit of energy (this parameter

is in good agreement between different approaches, see Tab. S2). For moderate changes in the magnetic parameters, the electromagnon energies are approximately linear functions of the variation, showing that small changes in parameters lead to small changes in the energies (up to 10% for variation in J_3 or K up to 20%). In particular, our calculated values of the electromagnon energies of 3.93 meV and 4.30 meV compare well with the experimental values of 4.09 meV and 4.51 meV, and would be even lower with the HSE parameters.

Based on these considerations, we are confident that our model provides a good description of the electromagnons and the associated ferroelectric properties, as validated by comparisons to both our DFT data and experiments.

Connection to the transient SHG signal. To connect the electromagnon modes to the observed modulation of the SHG signal, we first perform a symmetry analysis of how the magnon modes modulate the electric polarization and optical susceptibility tensor. Assuming that the amplitudes of the spin precessions are small enough, a given physical observable $\mathcal{A}$ can be expanded as

$$\mathcal{A}(t) = \mathcal{A}_{\text{eq}} + a_e m_e(t) + a_o m_o(t) + \mathcal{O}(m^2), \tag{9}$$

where $\mathcal{A}_{\text{eq}}$ is the value of the observable in equilibrium, and m_e and m_o denote the amplitudes of the C_2-even and C_2-odd magnon modes, respectively. The symmetry of the equilibrium system enforces that both a_e and a_o are invariant under a C_2 transformation and, by acting with this transformation on both sides of the equation, we see that $a_o = 0$ for a C_2-even observable while $a_e = 0$ for a C_2-odd observable.

As above, we define the x-axis to lie along the in-plane spiral wave vector, and the y-axis to be along the electric polarization. Thus, the y-axis is parallel to the C_2-axis. Therefore, since the x- and y-components of the polarization are respectively odd and even under the C_2 transformation, we expect the odd magnon mode EM_o to modulate the x-component of the transient electric polarization, and the even magnon mode EM_e to modulate the y-component of the transient electric polarization. We also expect that out of the six independent in-plane components of the optical susceptibility tensor, the C_2-even ones $\chi^{(2)}_{xxy}$, $\chi^{(2)}_{yxx}$ and $\chi^{(2)}_{yyy}$ will be modulated by EM_e, while the three C_2-odd ones $\chi^{(2)}_{yyx}$, $\chi^{(2)}_{xyy}$ and $\chi^{(2)}_{xxx}$ will be modulated by EM_o. These symmetry considerations are in good agreement with the modulation patterns observed in our experiments.

To provide a more quantitative analysis of the transient modulation of the SHG signal, we consider the response to a time-dependent external electric field described by the Hamiltonian

$$H_{\text{ext}} = \mathbf{A}(t) \cdot \hat{\mathbf{J}}. \tag{10}$$

Here, $\mathbf{A}(t) = (\mathbf{E}_0/\omega)\cos(\omega t)$ is the electromagnetic vector potential for an electric field with strength $\mathbf{E}_0$ and angular frequency ω, and the current operator $\hat{\mathbf{J}}$ is

$$\hat{\mathbf{J}} = \mathcal{J}\sum_\sigma [\chi_{1\sigma} p^\dagger_{1\sigma} d_1 \hat{\mathbf{e}}_1 + \chi_{2\sigma} p^\dagger_{2\sigma} d_2 \hat{\mathbf{e}}_2 + H.c.], \tag{11}$$

where $\mathcal{J}$ is the current integral and $\hat{\mathbf{e}}_1$ and $\hat{\mathbf{e}}_2$ are unit vectors along the Ni-I bonds shown

in Fig. S6. The optical response to second order in the perturbation H_{ext}, calculated via the second-order Kubo formula, provides the time-dependent polarization

$$\Delta\mathbf{P}^{(2)}(t) = -\int_{t_1}^{t}\int_{t_2}^{t_1} dt_1 dt_2 \langle [[\hat{\mathbf{P}}(t), H_{\text{ext}}(t_1)], H_{\text{ext}}(t_2)]\rangle, \tag{12}$$

where the operators $\hat{\mathbf{P}}(t)$ and $H_{\text{ext}}(t)$ are both in the Heisenberg picture with respect to the static Hamiltonian H_{cluster}. Treating again H_{so} as a perturbation to H_0, the polarization is given to leading order by

$$\begin{aligned}\Delta\mathbf{P}^{(2,1)}(t) = \frac{\lambda t \mathcal{J}^2 \mathcal{I}_1}{\omega^3\Delta^2(\Delta-\omega)}[\hat{\mathbf{e}}_3\cdot(\mathbf{S}_1\times\mathbf{S}_2)](\cos(2\omega t)\mathbf{E}_0\mathbf{E}_0) \qquad (13)\\ :[(\hat{\mathbf{e}}_1\hat{\mathbf{e}}_1\hat{\mathbf{e}}_1+\hat{\mathbf{e}}_2\hat{\mathbf{e}}_2\hat{\mathbf{e}}_2)+\frac{3}{2}(\hat{\mathbf{e}}_1\hat{\mathbf{e}}_2\hat{\mathbf{e}}_2+\hat{\mathbf{e}}_2\hat{\mathbf{e}}_1\hat{\mathbf{e}}_1)+\frac{3}{4}(\hat{\mathbf{e}}_1\hat{\mathbf{e}}_1\hat{\mathbf{e}}_2+\hat{\mathbf{e}}_2\hat{\mathbf{e}}_2\hat{\mathbf{e}}_1+\hat{\mathbf{e}}_1\hat{\mathbf{e}}_2\hat{\mathbf{e}}_1+\hat{\mathbf{e}}_2\hat{\mathbf{e}}_1\hat{\mathbf{e}}_2)].\end{aligned}$$

From this equation, we can identify the second-order optical susceptibility tensor,

$$\begin{aligned}\boldsymbol{\chi} = \frac{\lambda t \mathcal{J}^2 \mathcal{I}_1}{\omega^3\Delta^2(\Delta-\omega)}[\hat{\mathbf{e}}_3\cdot(\mathbf{S}_1\times\mathbf{S}_2)] \qquad (14)\\ \times[(\hat{\mathbf{e}}_1\hat{\mathbf{e}}_1\hat{\mathbf{e}}_1+\hat{\mathbf{e}}_2\hat{\mathbf{e}}_2\hat{\mathbf{e}}_2)+\frac{3}{2}(\hat{\mathbf{e}}_1\hat{\mathbf{e}}_2\hat{\mathbf{e}}_2+\hat{\mathbf{e}}_2\hat{\mathbf{e}}_1\hat{\mathbf{e}}_1)+\frac{3}{4}(\hat{\mathbf{e}}_1\hat{\mathbf{e}}_1\hat{\mathbf{e}}_2+\hat{\mathbf{e}}_2\hat{\mathbf{e}}_2\hat{\mathbf{e}}_1+\hat{\mathbf{e}}_1\hat{\mathbf{e}}_2\hat{\mathbf{e}}_1+\hat{\mathbf{e}}_2\hat{\mathbf{e}}_1\hat{\mathbf{e}}_2)].\end{aligned}$$

Similarly to the case of the electric polarization, the total susceptibility is obtained by summing over all nearest-neighbor bonds.

Although the above calculation was performed in the velocity gauge, the light-matter coupling (in the dipole approximation) can equivalently be written in the length gauge as

$$H_{\text{ext}} = \mathbf{E}(t)\cdot\hat{\mathbf{P}}. \tag{15}$$

This form of the light-matter coupling explains the large SHG signal observed below the ordering temperature of the equilibrium system, where the electric polarization acquires a finite expectation value $\langle\hat{\mathbf{P}}\rangle = \mathbf{P}_0$.

Supplementary Note 3: Microscopic source terms for SHG

In magnetic materials, several forms of spatial-inversion symmetry ($\mathcal{P}$) breaking could give rise to different second-order nonlinear responses. Here, we explore the potential contribution of these sources to the SHG signals observed in our NiI_2 measurements. This discussion also aims to contribute to the current debate on the origin of the SHG signal at our photon energy[2,7,17–19]. In all the SHG analysis to follow, we will use the x, y, z coordinate system from the main text (see Fig. 1c) and consider light beams propagating along the out-of-plane direction z.

From the wave equation, the source term for the nonlinear optical response $\mathbf{S}(t)$ can be expressed in terms of the induced nonlinear polarization $\mathbf{P}^{NL}$ and magnetization $\mathbf{M}^{NL}$ as

$$\mathbf{S}(t) = \mu_0 \left(\frac{\partial^2 \mathbf{P}^{NL}}{\partial t^2} + \nabla \times \frac{\partial \mathbf{M}^{NL}}{\partial t} \right), \tag{16}$$

where μ_0 is the vacuum magnetic permeability. For magnetic materials with broken $\mathcal{P}$ symmetry, the second-order nonlinear signal consists of two leading-order responses. In the plane-wave approximation with angular frequency ω, these can be written in the component-wise amplitudes of the nonlinear polarization $P_i^{NL}(2\omega)$ and magnetization $M_i^{NL}(2\omega)$ as (1) an electric dipole (ED) response of the form[20]

$$P_i^{NL}(2\omega) = \epsilon_0 \left(\chi_{ijk}^{(i,eee)} + \chi_{ijk}^{(c,eee)} \right) E_j(\omega) E_k(\omega), \tag{17}$$

and (2) a magnetic dipole (MD) response of the form[21]

$$M_i^{NL}(2\omega) = \epsilon_0 \frac{c}{n} \chi_{ijk}^{(c,mee)} E_j(\omega) E_k(\omega), \tag{18}$$

where ϵ_0 is the vacuum dielectric permittivity, c is the speed of light, n is the index of refraction at the angular frequency ω, and $E_i(\omega)$ is the component amplitude of the probe electric field. The second-order nonlinear optical susceptibilities $\chi_{ijk}^{(2)}$ are categorized as either i-type (time-invariant, representing crystalline asymmetry) or c-type (time-variant, representing the magnetic ordering).[20] In our experiments, both the fundamental and second harmonic probe photon energies are off-resonant with d-d or other parity-even transitions (see Extended Data Fig. 1 in the main text), signifying the absence of resonant enhancement for the MD pathway and making the MD response negligible compared to the ED response.[20]

Therefore, the main components of the SHG response are the ED terms $\chi_{ijk}^{(i,eee)}$ and $\chi_{ijk}^{(c,eee)}$ induced by the inversion-symmetry-broken ground state. The i-type SHG stems from the electric polarization, $\chi_{ijk}^{(i,eee)} \propto \boldsymbol{\mathcal{P}}_{FE}$, which reduces the crystalline point group to 2. In contrast, the c-type SHG originates from the antiferromagnetic order, $\chi^{(c,eee)} \propto \mathbf{L}$, which reduces the magnetic point group to either $21'$ or 1 depending on the treatment of the incommensurate spin-spiral[19]. Assuming the former $21'$ symmetry, the c-type SHG must necessarily be zero due to the gray magnetic point group[22], and hence the SHG signal can be ascribed to an i-type SHG of ferroelectric origin. If we instead take the latter 1 magnetic point group, then a c-type SHG is allowed in addition to the i-type SHG from the electric polarization. Assuming the induced $\boldsymbol{\mathcal{P}}_{FE}$ along y, the SHG arising from the latter will manifest at $\chi_{xxy}^{(i,eee)}$, $\chi_{yxx}^{(i,eee)}$, and $\chi_{yyy}^{(i,eee)}$, while the SHG arising from the spin spiral order will manifest at $\chi_{xxy}^{(c,eee)}$, $\chi_{yxx}^{(c,eee)}$, $\chi_{yyy}^{(c,eee)}$, $\chi_{yyx}^{(c,eee)}$,

$\chi_{xyy}^{(c,eee)}$, and $\chi_{xxx}^{(c,eee)}$. In this formulation, both types of symmetry breaking could, in principle, contribute to the overall SHG signal. However, we argue that the nonlinear response from the dipolar order still dominates in our experimental conditions.

One reason is the size of the observed nonlinear optical susceptibility in NiI_2 ($|\chi_{22}^{(2)}| \approx 20$ pm/V, see Supplementary Note 4), which is comparable to those from traditional ferroelectrics devoid of magnetic order, such as $BaTiO_3$ ($|\chi_{31}^{(2)}| = 36$ pm/V) and $LiNbO_3$ ($|\chi_{33}^{(2)}| = 69$ pm/V).[23] Apart from a few exceptions[24], the nonlinear optical susceptibility arising from magnetic ordering is substantially weaker than that originating from crystal asymmetry due to the requirement of higher-order effects (*e.g.*, spin-orbit coupling) to break parity symmetry in the electronic system[25,26]. In the same spirit, comparable multiferroic materials, including $MnWO_4$[27], $DyMn_2O_5$ [28], hexagonal $RMnO_3$ (R = Y, Ho, etc.)[20,29,30], exhibit predominant i-type SHG responses resulting from the material polarization outside of resonance with a d-d transition.

Another reason is the anisotropic SHG response. Significant contributions from both ferroelectric and spin-spiral orders would lead to an anisotropic response containing a mixture of $\chi_{ijk}^{(i,eee)}$ and $\chi_{ijk}^{(c,eee)}$ components, resulting in a loss of mirror symmetry in the polarimetry patterns. However, the experimentally observed SHG anisotropy exhibits a clear reflection symmetry and can be accurately assigned to a single ferroelectric domain with point group 2, using only the $\chi_{xxy}^{(i,eee)}$, $\chi_{yxx}^{(i,eee)}$, and $\chi_{yyy}^{(i,eee)}$ elements (see Fig. S9 and Table S5).

Finally, we note that should the magnetic point group be assigned to $21'$, the system would allow a magnetic SHG of i-type, $\chi_{ijk}^{(i,eee)} \propto \mathbf{L}^2$.[22] However, since $\boldsymbol{\mathcal{P}}_{FE} \propto \mathbf{L}^2$, such an SHG signal would be indistinguishable from the SHG induced by the electric polarization.

In combination, these factors strongly suggest that the observed SHG signal originates from an ED mechanism arising from the ferroelectric order, consistent with previous interpretations of the SHG signal from NiI_2[2,17]. This assignment is further supported by the observation of a $\pi/2$ phase shift between the tr-SHG and tr-RKerr signals observed in the main text.

Supplementary Note 4: Modelling of SHG Data

In this section, we report the derivation of the SHG models used to analyze the static and tr-SHG signals shown in the main text. We then utilize these models to extract the static second-order susceptibility and the dynamic susceptibility modulations induced by both electromagnon modes.

Static SHG. At normal incidence ($k_\omega, k_{2\omega}||z$), the most general second-order nonlinear polarization, $\mathbf{P}(2\omega)$, induced by an incoming electric field, $\mathbf{E}(\omega)$, is:

$$P_i = \epsilon_0 \sum_{jk} \chi^{(2)}_{ijk} E_j E_k, \text{with} \tag{19}$$

$$P_x = \epsilon_0[\chi^{(2)}_{xxx} E_x E_x + \chi^{(2)}_{xxy} E_x E_y + \chi^{(2)}_{xyx} E_y E_x + \chi^{(2)}_{xyy} E_y E_y]$$
$$P_y = \epsilon_0[\chi^{(2)}_{yxx} E_x E_x + \chi^{(2)}_{yxy} E_x E_y + \chi^{(2)}_{yyx} E_y E_x + \chi^{(2)}_{yyy} E_y E_y],$$

where $\chi^{(2)}_{ijk}$ is the third-rank nonlinear optical susceptibility tensor. Note that the frequencies ω have been omitted for simplicity. For a quasi-two-dimensional material with point group 2, *i.e.*, C_2 symmetry, there are 4 non-vanishing tensors elements assuming a two-fold axis parallel to y (crystallographic [010] axis) given by $\{xxy, xyx, yxx, yyy\}$. By applying the intrinsic permutation symmetry appropriate for SHG, $\chi^{(2)}_{xxy} = \chi^{(2)}_{xyx}$, we have

$$P_x = 2\epsilon_0 \chi^{(2)}_{xxy} E_x E_y \tag{20}$$
$$P_y = \epsilon_0[\chi^{(2)}_{yxx} E_x E_x + \chi^{(2)}_{yyy} E_y E_y],$$

Decomposing the electric polarization into azimuthal fields yields

$$P_x(\phi) = \epsilon_0 E^2[2\chi^{(2)}_{xxy} \cos\phi \sin\phi] \tag{21}$$
$$P_y(\phi) = \epsilon_0 E^2[\chi^{(2)}_{yxx} \cos^2(\phi) + \chi^{(2)}_{yyy} \sin^2(\phi)],$$

where ϕ is the azimuthal angle measured from the x-axis. Writing out the parallel- and cross-polarized SHG signals gives

$$P_{||}(\phi) = P_x(\phi)\cos\phi + P_y(\phi)\sin\phi \tag{22}$$
$$= \epsilon_0 E^2[(2\chi^{(2)}_{xxy} + \chi^{(2)}_{yxx})\sin\phi + (\chi^{(2)}_{yyy} - 2\chi^{(2)}_{xxy} - \chi^{(2)}_{yxx})\sin^3\phi]$$
$$P_{\perp}(\phi) = -P_x(\phi)\sin\phi + P_y(\phi)\cos\phi \tag{23}$$
$$= \epsilon_0 E^2[(\chi^{(2)}_{yyy} - 2\chi^{(2)}_{xxy})\cos\phi - (\chi^{(2)}_{yyy} - 2\chi^{(2)}_{xxy} - \chi^{(2)}_{yxx})\cos^3\phi].$$

Suppose the electric polarization is along x instead. The nonlinear polarization can then be obtained by making the replacements $x \to y$, $y \to x$ and $\phi \to \phi + \frac{\pi}{2}$, yielding

$$P'_{||}(\phi) = \epsilon_0 E^2[(2\chi^{(2)}_{yyx} + \chi^{(2)}_{xyy})\cos\phi + (\chi^{(2)}_{xxx} - \chi^{(2)}_{xyy} - 2\chi^{(2)}_{yyx})\cos^3\phi] \tag{24}$$
$$P'_{\perp}(\phi) = \epsilon_0 E^2[(2\chi^{(2)}_{yyx} - \chi^{(2)}_{xxx})\sin\phi + (\chi^{(2)}_{xxx} - \chi^{(2)}_{xyy} - 2\chi^{(2)}_{yyx})\sin^3\phi].$$

Similarly, if the electric polarization is along some general axis, the total nonlinear polarization is the combination

$$P_{||}^{tot}(\phi) = P_{||}(\phi) + P'_{||}(\phi)$$
$$P_{\perp}^{tot}(\phi) = P_{\perp}(\phi) + P'_{\perp}(\phi). \tag{25}$$

In equilibrium, we take the electric polarization from a single domain to be along y and fit the static SHG signal intensity to Eqs. 22 and 23. The resulting fits, plotted in Fig. S9, show excellent agreement with the experimental data, with the corresponding nonlinear optical susceptibilities given in Table S3.

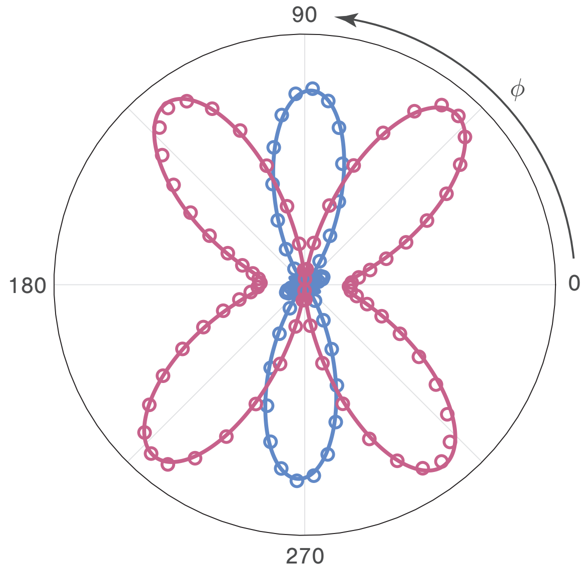

Fig. S9 | Static anisotropic SHG model. The experimental static SHG polar pattern (*circles*) is plotted alongside fits (*solid lines*) for both parallel- (*blue*) and cross- (*red*) polarization configurations. The C_2-axis (y-axis) is along $\phi = 90°$.

$\chi^{(2)}_{xxy}$	-14.6 pm/V
$\chi^{(2)}_{yxx}$	8.6 pm/V
$\chi^{(2)}_{yyy}$	19.7 pm/V

Table S3 | Static second-order nonlinear optical susceptibilities calculated from the calibrated SHG power, a probe pulse duration of 270 fs, a beam size of 1 μm and an optical penetration depth of 20 nm at $\hbar\omega$ = 2.4 eV.

Time-resolved SHG. Assume that the elements of the nonlinear optical susceptibility tensor are proportional to the component of the instantaneous electric polarization allowed by symmetry

$$\chi^{(2)}_{xxy}(t), \chi^{(2)}_{yxx}(t), \chi^{(2)}_{yyy}(t) \propto \mathcal{P}^y(t) \tag{26}$$
$$\chi^{(2)}_{yyx}(t), \chi^{(2)}_{xyy}(t), \chi^{(2)}_{xxx}(t) \propto \mathcal{P}^x(t). \tag{27}$$

This means that the nonlinear polarizations can be separated into azimuthal components $V(\phi)$ and temporal components $\mathcal{P}(t)$ as

$$P_{||}^{tot}(\phi,t) = V_{||}(\phi)\mathcal{P}_y(t) + V'_{||}(\phi)\mathcal{P}_x(t) \tag{28}$$

$$P_{\perp}^{tot}(\phi,t) = V_{\perp}(\phi)\mathcal{P}_y(t) + V'_{\perp}(\phi)\mathcal{P}_x(t), \tag{29}$$

Empirically, the time-dependent electric polarization along y can be written as

$$\mathcal{P}_y(t) = \mathcal{P}_0 + \Delta\mathcal{P}_y(t), \tag{30}$$

which includes the EME$_e$ electromagnon, $\Delta\mathcal{P}_y(t)$, and the the initial polarization, $\mathcal{P}_0$. The electric polarization along x includes only a contribution from the EM_o electromagnon:

$$\mathcal{P}_x(t) = \Delta\mathcal{P}_x(t).$$

The associated time-dependent anisotropic nonlinear polarizations are

$$P(\phi,t) = \mathcal{P}_0(\phi) + \Delta\mathcal{P}_y(\phi,t) \tag{31}$$

$$= V(\phi)(\mathcal{P}_0 + \Delta\mathcal{P}_y(t)) \tag{32}$$

$$P'(\phi,t) = \Delta\mathcal{P}_x(\phi,t) \tag{33}$$

$$= V'(\phi)\Delta\mathcal{P}_x(t). \tag{34}$$

Finally, we can write the time-dependent SHG intensity as

$$I(\phi,t) = |P^{tot}(\phi,t)|^2 \tag{35}$$

$$= |P(\phi,t) + P'(\phi,t)|^2 \tag{36}$$

$$\begin{aligned} &= \mathcal{P}_0(\phi)^2 + 2\mathcal{P}_0(\phi)[\Delta\mathcal{P}_x(\phi,t) + \Delta\mathcal{P}_y(\phi,t)] + \\ &\quad \Delta\mathcal{P}_x(\phi,t)^2 + \Delta\mathcal{P}_y(\phi,t)^2 + 2\Delta\mathcal{P}_x(\phi,t)\Delta\mathcal{P}_y(\phi,t). \end{aligned} \tag{37}$$

In the limit of weak electromagnon modulation, *i.e.*, $\mathcal{P}_0 \gg \Delta\mathcal{P}_x(t), \Delta\mathcal{P}_y(t)$, heterodyning of the electromagnon signal with the SHG background results in an overall background-free signal modulation of:

$$\Delta I(\phi,t) = I(\phi,t) - \mathcal{P}_0(\phi)^2 \tag{38}$$

$$= 2\mathcal{P}_0(\phi)[\Delta\mathcal{P}_x(\phi,t) + \Delta\mathcal{P}_y(\phi,t)], \tag{39}$$

which yields the following expressions for the time-dependent SHG intensity:

$$\begin{aligned} \Delta I_{||}(\phi,t) = 2\mathcal{P}_{0,||}(\phi)[&(2\Delta\chi^{(2)}_{xxy} + \Delta\chi^{(2)}_{yxx})\sin\phi + (\Delta\chi^{(2)}_{yyy} - 2\Delta\chi^{(2)}_{xxy} - \Delta\chi^{(2)}_{yxx})\sin^3\phi + \\ &(2\Delta\chi^{(2)}_{yyx} + \Delta\chi^{(2)}_{xyy})\cos\phi + (\Delta\chi^{(2)}_{xxx} - \Delta\chi^{(2)}_{xyy} - 2\Delta\chi^{(2)}_{yyx})\cos^3\phi] \end{aligned} \tag{40}$$

$$\begin{aligned} \Delta I_{\perp}(\phi,t) = 2\mathcal{P}_{0,\perp}(\phi)[&(\Delta\chi^{(2)}_{yyy} - 2\Delta\chi^{(2)}_{xxy})\cos\phi - (\Delta\chi^{(2)}_{yyy} - 2\Delta\chi^{(2)}_{xxy} - \Delta\chi^{(2)}_{yxx})\cos^3\phi + \\ &(2\Delta\chi^{(2)}_{yyx} - \Delta\chi^{(2)}_{xxx})\sin\phi + (\Delta\chi^{(2)}_{xxx} - \Delta\chi^{(2)}_{xyy} - 2\Delta\chi^{(2)}_{yyx})\sin^3\phi]. \end{aligned} \tag{41}$$

We then proceed to fit this model to the experimentally measured tr-SHG signal. To focus exclusively on the coherent electromagnons' oscillatory response, we eliminate the incoherent

background from the raw data. This is achieved by fitting the temporal traces at each probe polarization angle to a double-exponential decay. Due to the challenge of the global fit in accurately capturing the incoherent background at early time delays for all probe polarizations, we only consider the oscillatory signal starting from $\sim$2 ps.

Single-oscillator model. To account for the simplest scenario, we first model the signal with a generalized single harmonic oscillator whose components can be written as:

$$\begin{aligned}
\Delta\chi^{(2)}_{xxy}(t) &= A_{xxy}e^{-\gamma t}\cos\left(\omega t + \Phi\right) \\
\Delta\chi^{(2)}_{yxx}(t) &= A_{yxx}e^{-\gamma t}\cos\left(\omega t + \Phi\right) \\
\Delta\chi^{(2)}_{yyy}(t) &= A_{yyy}e^{-\gamma t}\cos\left(\omega t + \Phi\right) \\
\Delta\chi^{(2)}_{yyx}(t) &= A_{yyx}e^{-\gamma t}\cos\left(\omega t + \Phi\right) \\
\Delta\chi^{(2)}_{xyy}(t) &= A_{xyy}e^{-\gamma t}\cos\left(\omega t + \Phi\right) \\
\Delta\chi^{(2)}_{xxx}(t) &= A_{xxx}e^{-\gamma t}\cos\left(\omega t + \Phi\right),
\end{aligned} \tag{42}$$

where A_i, γ, ω, and Φ are the amplitude, decay constant, angular frequency, and initial phase of the respective oscillations. The corresponding fits to the experimental data and residual signals are shown in Fig. S10, with the fitted parameter values shown in Table S4. The results reproduce the strong 0.97 THz (4.01 meV) oscillations $||$ C_2 axis in both parallel- and cross-polarized detection configurations, *i.e.*, around $\phi = 90°, 270°$, which we can assign to the C_2-even electromagnon. However, the lower frequency oscillations observed most prominently in the perpendicular polarization near $\phi = 0°, 180°$ are not captured by this model, as can be seen in the significant oscillation in the residual. This indicates that a single oscillator cannot explain the complex anisotropic oscillatory features in the tr-SHG signal. Note that the oscillation represented by this model does not need to obey the underlying symmetry of the lattice, so it is notable that the results are consistent with a dominant C_2-even oscillation.

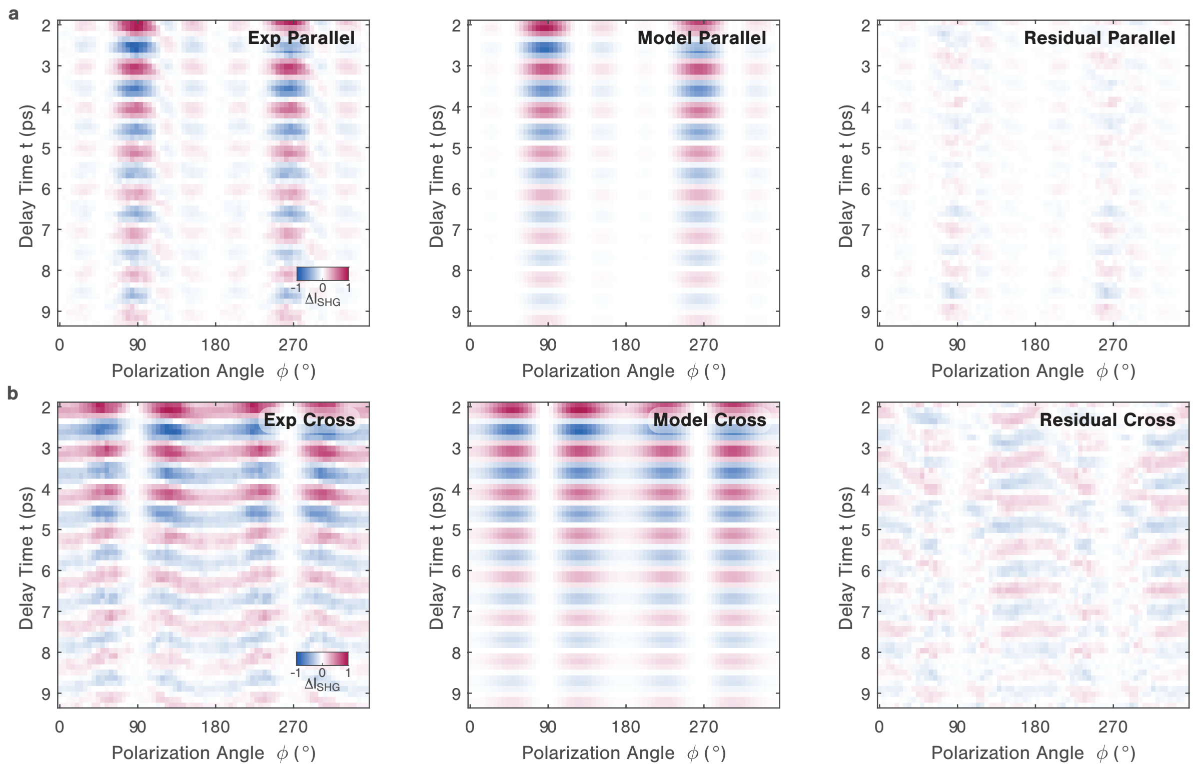

Fig. S10 | Single-oscillator tr-SHG model. Results obtained from fitting the experiment tr-SHG signal in the **a,** parallel- and **b,** cross-polarized detection channels with a single-oscillator model. The corresponding fitting parameters are reported in Table S4.

ijk	A_{ijk} (fm/V)	γ (ps^{-1})	$\omega/2\pi$ (THz)	Φ/π (rad)
xxy	-89	0.28	0.98	-0.03
yxx	46			
yyy	96			
yyx	11			
xyy	20			
xxx	14			

Table S4 | Single-oscillator tr-SHG fitting parameters.

Two-oscillator model. To reproduce all the significant features of the tr-SHG signal, we now turn to a two-oscillator model. As the previous single-oscillator model suggests, the dominant oscillation originates from a predominantly C_2-even mode. Thus, it is natural to choose a pair of C_2-even and C_2-odd oscillations with angular frequencies ω_e and ω_o respectively. By doing so and assuming that the two modes are perfectly orthogonal, we do not observe perfect agreement with the data due to the enforced symmetry of the C_2-odd oscillations. Instead, we expect some symmetry-breaking upon photoexcitation, as evidenced by the slight asymmetry in the induced incoherent anisotropic tr-SHG background. Through an iterative fit of the time-resolved SHG data, we establish this angle to be 25°. Assuming that the C_2-odd mode has its principal axis rotated by 25° defined as the x' and y' axes, we can then fit the data with a model

of the form:

$$\begin{aligned}\Delta\chi^{(2)}_{xxy}(t) &= A_{xxy}e^{-\gamma_e t}\cos\left(\omega_e t+\Phi\right)\\ \Delta\chi^{(2)}_{yxx}(t) &= A_{yxx}e^{-\gamma_e t}\cos\left(\omega_e t+\Phi\right)\\ \Delta\chi^{(2)}_{yyy}(t) &= A_{yyy}e^{-\gamma_e t}\cos\left(\omega_e t+\Phi\right)\\ \Delta\chi^{(2)}_{y'y'x'}(t) &= A_{y'y'x'}e^{-\gamma_o t}\cos\left(\omega_o t+\Phi\right)\\ \Delta\chi^{(2)}_{x'y'y'}(t) &= A_{x'y'y'}e^{-\gamma_o t}\cos\left(\omega_o t+\Phi\right)\\ \Delta\chi^{(2)}_{x'x'x'}(t) &= A_{x'x'x'}e^{-\gamma_o t}\cos\left(\omega_o t+\Phi\right).\end{aligned} \tag{43}$$

To apply this model, we perform a preliminary fit of the raw time traces for each probe polarization using a damped sinusoidal function. Afterward, we conduct the full fit by fixing the phase of the oscillations to the average phase obtained through the pre-fits. The results of the fit and the residuals are shown in Fig. S11, while the fitting parameters can be found in Table S5. Within this model, the frequencies of the two electromagnon modes are 0.97 THz (4.01 meV) and 0.93 THz (3.85 meV). As noted in the main text and seen in the greatly suppressed residual signal, this fixed-phase two-oscillator model accurately reproduces the major oscillatory features in the tr-SHG data. However, we note that allowing the oscillations' phase to vary freely yields an almost identical phase. Letting the phase of the two oscillators vary independently (*e.g.*, $\Phi_o \neq \Phi_e$) leads instead to unphysical results, where the two oscillators have the same frequency but dramatically different phases and damping rates.

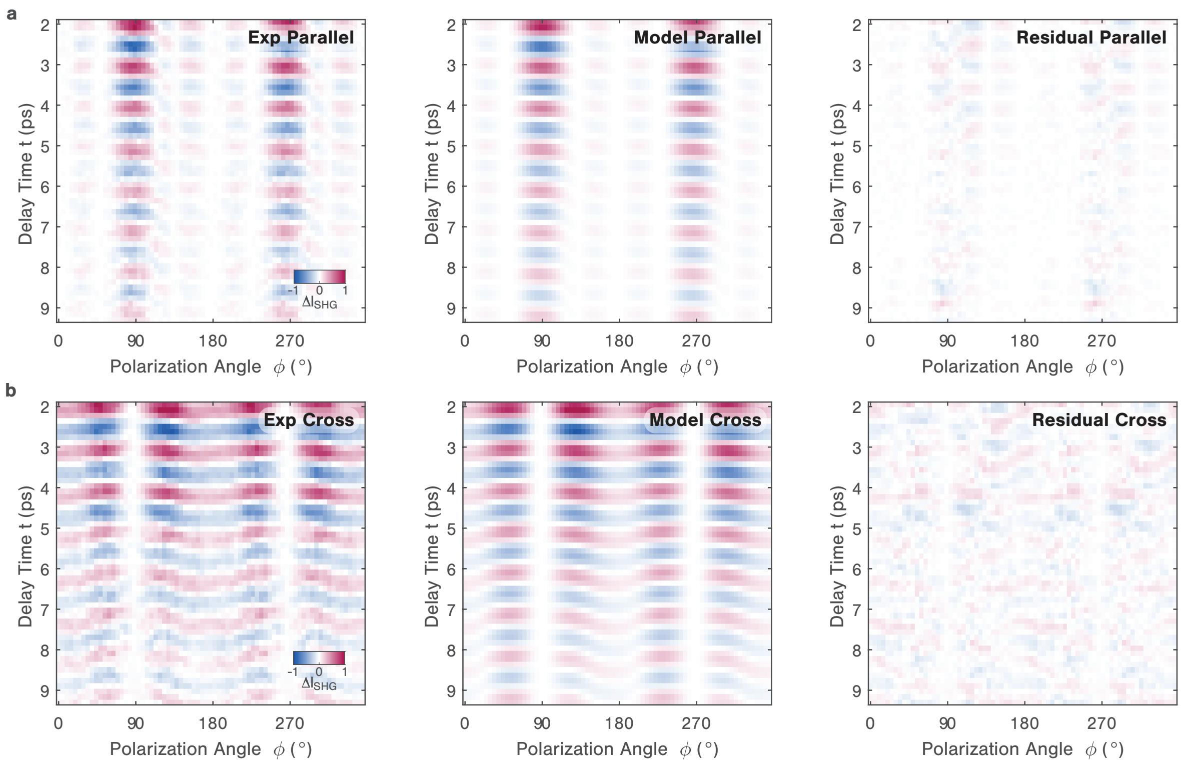

Fig. S11 | Two-oscillator tr-SHG model. Results obtained from fitting the experimental tr-SHG signal in the **a,** parallel- and **b,** cross-polarized detection channels with a two-oscillator model. Corresponding fitting parameters are reported in Table S5.

ijk	A_{ijk} (fm/V)	γ (ps^{-1})	$\omega/2\pi$ (THz)	Φ/π (rad)
xxy	-113	0.31	0.97	-0.02
yxx	31			
yyy	126			
$y'y'x'$	-26	0.10	0.93	
$x'y'y'$	-8.1			
$x'x'x'$	21			

Table S5 | Fitting parameters of the two-oscillator tr-SHG model.

Unconstrained oscillator model. Lastly, we note that an alternative, less constrained fitting procedure can be used to fit the above model to the tr-SHG data. The results of such a fitting procedure are presented in Figs. 3a and 3b of the main text, with the full parameters shown below in Table S6. Here, rather than fitting the overall anisotropic tr-SHG signal with a pair of oscillators, we first fit the time-dependent polar patterns obtained in both parallel- and cross-polarized detection configurations to Eqs. 40 and 41. Doing so yields a set of six time-dependent susceptibility modulations $\Delta\chi_i^{(2)}(t)$ which we then fit with damped oscillators of the form:

$$\Delta\chi_i^{(2)}(t) = A_i e^{-\gamma_i t} \cos(\omega_i t + \Phi). \tag{44}$$

With this method, we can identify a pair of oscillators that correspond to the higher frequency EM_e and lower frequency EM_o modes. Moreover, the observed amplitudes, decay, and frequencies match well with the two-oscillator model presented above. To retrieve reliable fitting parameters, we smooth the raw anisotropic tr-SHG data and fix the initial phase of the oscillators in the fits. Specifically, we first perform a free fit of the data and then use the average phase of the resulting fits (-0.12π rad).

ijk	A_{ijk} (fm/V)	γ (ps^{-1})	$\omega/2\pi$ (THz)	Φ/π (rad)
xxy	-92	0.33	0.98	-0.12
yxx	30	0.15	0.97	
yyy	95	0.32	0.98	
$y'y'x'$	-28	0.14	0.93	
$x'y'y'$	-14	0.14	0.90	
$x'x'x'$	30	0.22	0.93	

Table S6 | Fitting parameters of the unconstrained oscillator tr-SHG model.

Supplementary Note 5: Comparison between tr-RKerr and tr-SHG signals

Thus far, we have demonstrated that tr-SHG is an excellent tool for capturing the dynamical evolution of the electromagnon modes in NiI_2 through modulations of the electric polarization. In this section, we explore the use of a complementary tool, time-resolved reflective Kerr effect (tr-RKerr), to probe the corresponding evolution of the transient magnetization of the same electromagnons. In particular, we will: i) demonstrate the contrasting phase between the observed polarization and magnetization modulations; ii) extract the fluence-dependent Kerr rotation parameters for both electromagnons; and iii) calculate the relationship between the observed reflective Kerr rotation and the electromagnon density.

For tr-RKerr measurements, we employed a polar Kerr geometry and monitored the pump-induced polarization rotation of a linearly polarized probe reflected off a sample at normal incidence. By recording this light polarization rotation signal ($\Delta\theta_K$) as a function of pump-probe delay, we sought to measure the transient modulation of the out-of-plane magnetization due to the coherent electromagnon modes. For these tr-RKerr measurements, we chose the same pump and probe photon energies as in tr-SHG, *i.e.*, $\hbar\omega_{pump}$ = 1.13 eV, $\hbar\omega_{probe}$ = 1.20 eV, which enabled us to compare the two results (as shown below). For tr-SHG, we utilized an x-polarized probe, which selected the EM_o oscillation and allowed us to compare the phases of the two oscillations accurately. For tr-RKerr, we chose the same probe polarization along x, as this also minimized the incoherent background.

Figure S12 displays the tr-SHG and tr-RKerr signals obtained at several incident pump fluences on NiI_2. Both techniques yield traces with similar features. In all cases, the signals consist of a fast, resolution-limited response that is followed by a decaying incoherent background and coherent electromagnon oscillations. We then isolate the oscillatory response by plotting the background-subtracted tr-SHG and tr-RKerr signals and show the results in Fig. S13a,b. A visual inspection of the two signals reveals that the two techniques yield similar oscillatory dynamics that are characterized by a nearly monotonic decay, indicative of the oscillation of a single mode. We then fit this oscillation to a decaying sinusoid of the form

$$Ae^{-\gamma t}\cos(\omega t + \Phi), \tag{45}$$

where A is the amplitude, γ is the decay constant, ω is the angular frequency, and Φ is phase of the oscillator. These fits (*solid lines*) are plotted alongside the experimental data in Fig. S13a,b, with the oscillatory parameters shown in Fig. S14. The amplitude of the oscillations in both probes scales linearly with the incident pump fluence, indicating that our measurements are performed in a linear regime of pump-probe spectroscopy. The oscillations in both probes also share a common energy softening on the incident pump fluence, suggesting that the same electromagnon mode EM_o is coherently excited by the pump pulse. This scenario is further supported by the evolution of the decay constant, which is the same for both oscillations and linearly increases as a function of incident pump fluence. Such a trend can be explained by a combination of pure dephasing (*i.e.*, through mutual scattering processes on the same magnon branch) and population relaxation (*i.e.*, anharmonic decay via three/four-magnon scattering processes or charge carrier-mediated processes)[31–35]. Although both tr-SHG and tr-RKerr clearly show the evolution of the same coherent modes, a visual inspection of the oscillations reveals that the two detection schemes show contrasting initial phases. This behavior is confirmed by examining the fitted phase in Fig. S14 which exhibits a clear $\pi/2$ shift between the

probes. Notably, the same phase difference is seen in the calculated polarization and magnetization dynamics of the electromagnons as shown in Fig. S7 and discussed in Supplementary Note 2. Below, we will attribute this phase shift to assignments of the tr-SHG and tr-RKerr signals to modulations of the electric polarization and transient magnetization, respectively. Finally, it should be noted that neither probe sees substantial contributions from the higher frequency EM_e. For the x-polarized tr-SHG signal, this behavior can be explained by the fact that x is orthogonal to the polarization modulation induced by EM_e (*i.e.*, along y) and hence the SHG probe cannot detect it. Furthermore, calculations show that the EM_e mode exhibits no out-of-plane magnetization. Hence, no polar magneto-optical Kerr rotation would be seen in the tr-RKerr signal.

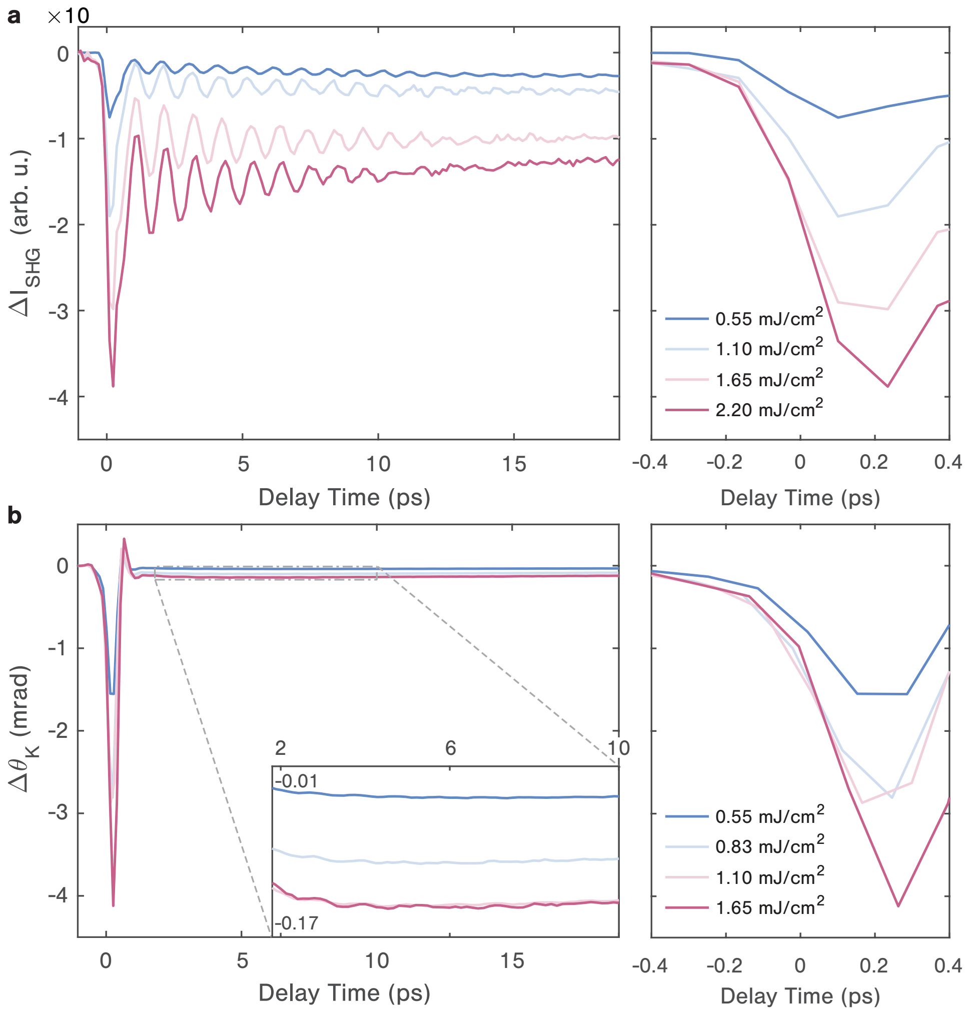

Fig. S12 | Fluence-dependent tr-SHG and tr-RKerr response. **a** Time-resolved SHG signal, ΔI_{SHG}, obtained from measurements of NiI_2 at several incident pump fluences. The signal shows coherent oscillations corresponding to the C_2-odd electromagnon, EM_o. **b** Time-resolved reflective Kerr rotation, $\Delta\theta_K$, obtained from measurements of NiI_2 at several incident pump fluences. The inset shows the weak oscillatory features in the Kerr rotation, which is otherwise dominated by a strong incoherent response. For both sets of measurements, time-zero was uniformly determined by fitting the initial coherent response with an error function and setting the inflection point as zero-delay. The right panels show the corresponding signals in the region near time-zero.

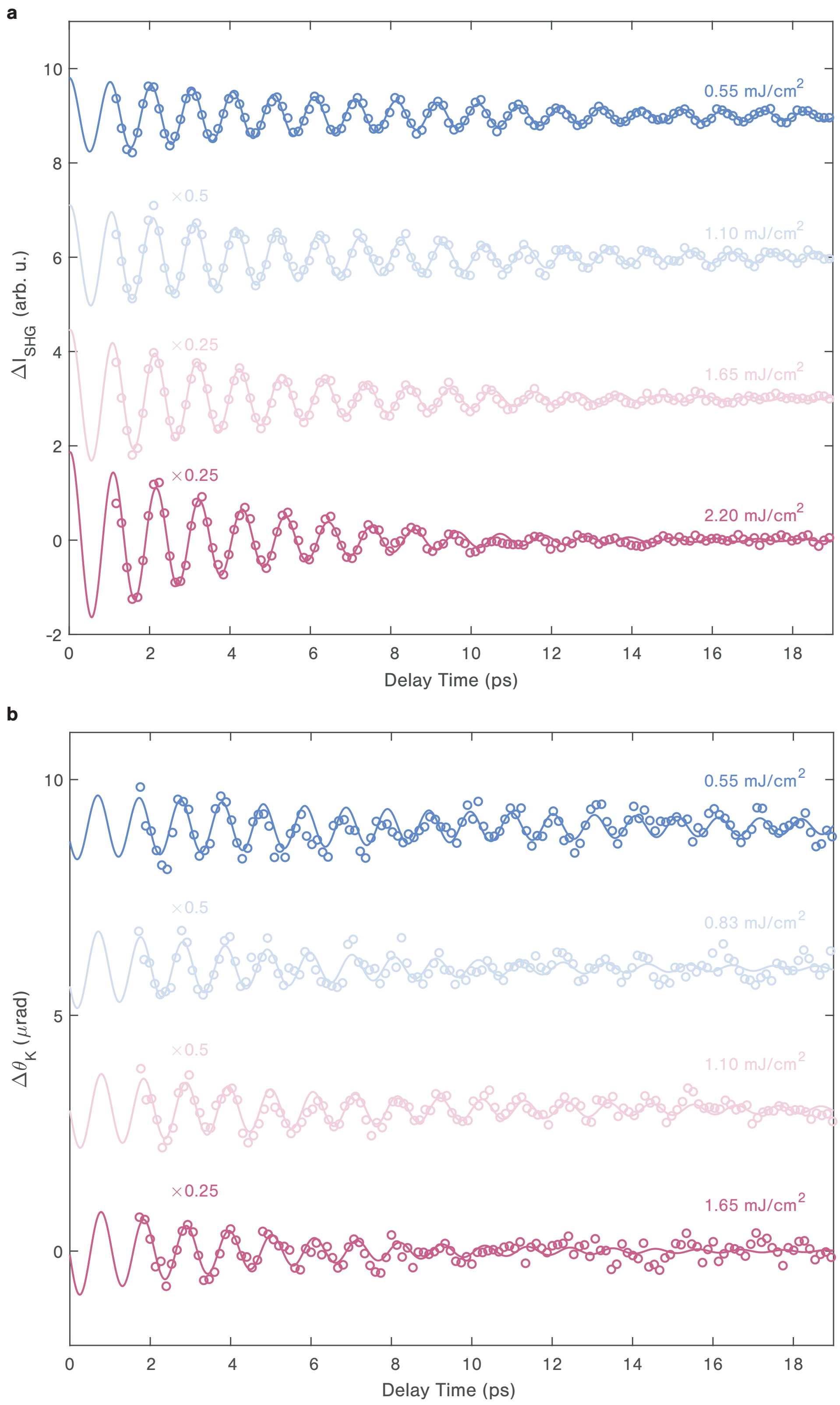

Fig. S13 | Electromagnon oscillations in tr-SHG and tr-RKerr. Background-subtracted time-resolved **a,** SHG and **b,** Kerr signals, for several incident pump fluences. Experimental data (*circles*) is shown alongside fits to one (*solid*, tr-SHG) and two (*solid*, tr-RKerr) damped oscillators of the form $Ae^{-\gamma t}\cos(\omega t + \Phi)$. For clarity, tr-SHG and tr-RKerr traces are offset by increments of 3 μV and 3 μrad, respectively.

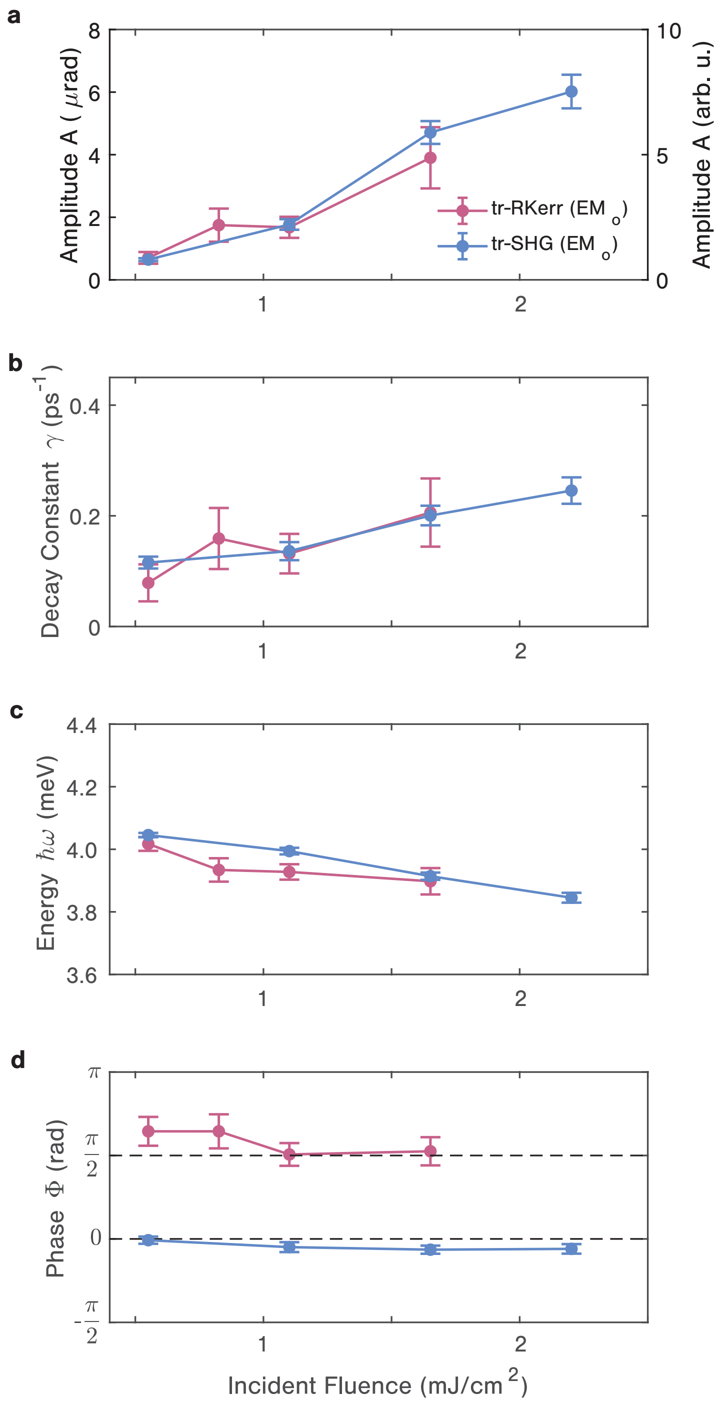

Fig. S14 | Fitted electromagnon oscillatory parameters at 2.4 K. Observed EM_o oscillatory parameters from both tr-SHG and tr-RKerr measurements: **a,** amplitude A, **b,** decay constant γ, **c,** energy $\hbar\omega$, and **d,** the initial oscillatory phases, Φ, obtained from the corresponding fits in Fig. S13 as a function of the incident pump fluence. All error bars show the 95% confidence intervals obtained from the fits.

Theoretical analysis of tr-RKerr data. In this section, we disentangle the relative contributions of polarization and magnetization dynamics in the reflective Kerr signal by performing a perturbative expansion with regard to the pump-induced dielectric responses. To begin, we consider the electric displacement $\mathbf{D}$ field in a biaxial magneto-optical crystal, which we write

in terms of the electric field $\mathbf{E}$ as

$$\begin{aligned}\mathbf{D} &= \epsilon_0 \left(\boldsymbol{\varepsilon} + i\mathbf{G}\right) \mathbf{E} \\ &= \epsilon_0 \left[\begin{pmatrix} \varepsilon_{xx} & \varepsilon_{xy} & \varepsilon_{xz} \\ \varepsilon_{yx} & \varepsilon_{yy} & \varepsilon_{yz} \\ \varepsilon_{zx} & \varepsilon_{zy} & \varepsilon_{zz} \end{pmatrix} + i \begin{pmatrix} 0 & -G_z & G_y \\ G_z & 0 & -G_x \\ -G_y & G_x & 0 \end{pmatrix} \right] \mathbf{E},\end{aligned}$$

where $\boldsymbol{\varepsilon}$ is the anisotropic permittivity tensor, $\mathbf{G}$ is the gyrotropic tensor, and ϵ_0 is the permittivity of free space. Choosing the axis $\{x, y, z\}$ to be the optical axes of the crystal, the permittivity tensor can be written as

$$\varepsilon_{ii} = \varepsilon_{ii}^0 + \Delta\varepsilon_{ii} \tag{46}$$

$$\varepsilon_{ij} = \Delta\varepsilon_{ij}, i \neq j, \tag{47}$$

where ε_{ii}^0 are components of the diagonal static permittivity tensor and $\Delta\varepsilon_{ij}$ are the pump-induced modulations of the permittivity. We now consider an electromagnetic wave at optical frequencies, propagating in the crystal with wavevector $\mathbf{k}||z$. The wave equation can be written as

$$\mathbf{k} \times \mathbf{k} \times \mathbf{E} + k_0^2 \left(\boldsymbol{\varepsilon} + i\mathbf{G}\right) \mathbf{E} = 0. \tag{48}$$

Taking into account that the anisotropy in ε only occurs in the xy-plane (due to the normal incidence of the probe beam), the equation above can be reduced to

$$k_0^2 \begin{pmatrix} \varepsilon_{xx} & \varepsilon_{xy} - iG_z \\ \varepsilon_{yx} + iG_z & \varepsilon_{yy} \end{pmatrix} \mathbf{E} = k_z^2 \mathbf{E}, \tag{49}$$

where $k_0 = 2\pi/\lambda_0$ and λ_0 is the wavelength of the incident light beam in vacuum. Thus, we see that $(k_z/k_0)^2$ is the eigenvalue for the total complex Hermitian permittivity tensor

$$\tilde{\boldsymbol{\varepsilon}} = \begin{pmatrix} \varepsilon_{xx} & \varepsilon_{xy} - iG_z \\ \varepsilon_{yx} + iG_z & \varepsilon_{yy} \end{pmatrix}. \tag{50}$$

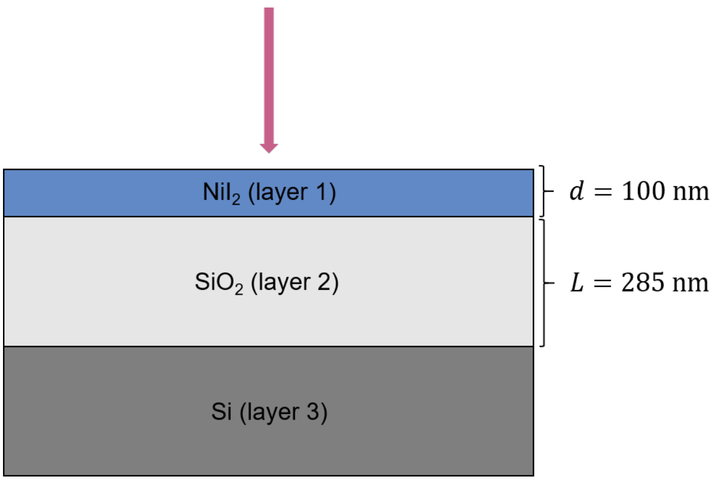

Fig. S15 | Schematic diagram of the sample-substrate system. A 100-nm-thick exfoliated NiI_2 sample was deposited on a 285-nm-thick SiO_2 layer that lied on a Si substrate.

The reflected probe light traversing this optical system will experience a complex polarization rotation originating from both the real ($\Delta\epsilon_{yx}$) and imaginary (G_z) components of the dielectric tensor's off-diagonal elements due to modulations of the C_2-odd electromagnon mode. To quantify this polarization rotation signal, we first write the transfer matrix of a trilayer optical system (see Fig. S15) consisting of a NiI_2 sample (with thickness d) placed on a layer of SiO_2 (with thickness L) and a Si substrate. We account for the pump-induced birefringence as

$$\mathcal{T}_{03} = T_{01}\mathcal{G}_1(d)T_{12}\mathcal{G}_2(L)T_{23},$$

where T_{ij} accounts for the boundary conditions of electromagnetic waves at the interface between layers i and j:

$$T_{ij} = \begin{pmatrix} \mathbf{1} & \mathbf{1} \\ \sqrt{\tilde{\varepsilon}_j} & -\sqrt{\tilde{\varepsilon}_j} \end{pmatrix}^{-1} \begin{pmatrix} \mathbf{1} & \mathbf{1} \\ \sqrt{\tilde{\varepsilon}_i} & -\sqrt{\tilde{\varepsilon}_i} \end{pmatrix}. \tag{51}$$

The bold $\mathbf{1}$ is the 2×2 identity matrix, $\tilde{\varepsilon}_i$ and $\tilde{\varepsilon}_j$ are the 2×2 Hermitian permittivity tensors for layer i and j, as defined in Eq. 50. The square root indicates the arithmetic square root for positive-definite matrices, *i.e.*, if $\tilde{\mathbf{B}} = \sqrt{\tilde{\mathbf{A}}}$ then $\tilde{\mathbf{B}}\tilde{\mathbf{B}} = \tilde{\mathbf{A}}$, where both $\tilde{\mathbf{A}}$ and $\tilde{\mathbf{B}}$ are positive-definite Hermitian matrices.

$\mathcal{G}_i(l)$ are propagation matrices accounting for the bidirectional traversal of light through layer i with thickness l:

$$\mathcal{G}_i(l) = \begin{pmatrix} e^{i2\pi\sqrt{\tilde{\varepsilon}_i}\frac{l}{\lambda_0}} & \mathbf{0} \\ \mathbf{0} & e^{-i2\pi\sqrt{\tilde{\varepsilon}_i}\frac{l}{\lambda_0}} \end{pmatrix}, \tag{52}$$

where the exponential and square root are also for matrices. Assuming that the incident, reflected, and transmitted light fields have strength $\mathbf{E_i}$, $\mathbf{E_r}$, and $\mathbf{E_t}$, we have

$$\mathcal{T}_{03}\begin{pmatrix} \mathbf{E_i} \\ \mathbf{E_r} \end{pmatrix} = \begin{pmatrix} \mathbf{E_t} \\ 0 \end{pmatrix}.$$

Given $\mathbf{E_i}$, we can solve for $\mathbf{E_t}$ and $\mathbf{E_r}$. In the experimental setup, the incident light has its polarization perpendicular to the C_2 axis, and thus $\mathbf{E_i} = (1,\, 0)^T$. Accordingly, the reflected Kerr signal is calculated by

$$\Delta\theta_K = \frac{|\mathbf{E_{r}}_x + \mathbf{E_{r}}_y|^2 - |\mathbf{E_{r}}_x - \mathbf{E_{r}}_y|^2}{|\mathbf{E_{r}}_x + \mathbf{E_{r}}_y|^2 + |\mathbf{E_{r}}_x - \mathbf{E_{r}}_y|^2}. \tag{53}$$

Using DFT calculations, we obtained the in-plane permittivity tensor of NiI_2 without pumping, which reads

$$\tilde{\varepsilon} = \begin{pmatrix} \varepsilon_{xx} & 0 \\ 0 & \varepsilon_{yy} \end{pmatrix}, \tag{54}$$

where $\varepsilon_{xx} \approx 6.87$ and $\varepsilon_{yy} \approx 6.98$. Note that, in our trilayer system, only layer 1 (*i.e.*, NiI_2) possesses any notable birefringence. We can see that, without pumping, the reflected light also polarizes along the x axis; thus, $\Delta\theta_K = 0$.

Through our DFT calculations, we also obtained the modulation of the permittivity tensor by EM_o, which reads

$$\Delta\tilde{\varepsilon} = \begin{pmatrix} 0 & \Delta\varepsilon_{xy} - iG_z \\ \Delta\varepsilon_{xy} + iG_z & 0 \end{pmatrix}, \tag{55}$$

where $\Delta\varepsilon_{xy} \approx 0.016\rho_{EM}$, $G_z \approx$ -0.010 ρ_{EM}, and ρ_{EM} is the electromagnon density (*i.e.*, the number of electromagnons per unit cell). In the limit of small electromagnon modulations, the reflective Kerr signal takes the form of

$$\Delta\theta_K \approx A_\varepsilon \Delta\varepsilon_{xy} + A_G G_z, \tag{56}$$

where A_ε and A_G are coefficients. After pumping, we create a coherent electromagnon density in the system, giving rise to a non-zero oscillating Kerr signal. Using the experimental parameters L = 285 nm, d = 100 nm, and the refraction indices of SiO_2 and Si at 1.20 eV ($n_b \approx$ 1.45 and $n_3 \approx$ 3.57), we have $|A_G| \gg |A_\epsilon|$ and $\Delta\theta_K \approx 0.0023\rho_{EM}$, where ρ_{EM} is the electromagnon density derived from the modulation amplitude of the Kerr angle. This electromagnon density will play a critical role in the estimate of the dynamical magnetoelectric coupling constant (see Supplementary Note 6). We also emphasize that, in our experimental geometry, the Kerr signal mainly stems from the gyrotropic contribution G_z. This is confirmed by manually setting $\Delta\varepsilon_{xy}$ = 0 in the calculations, as the resulting $\Delta\theta_K \approx 0.0025\rho_{EM}$ remains almost unchanged.

Phase of the oscillations in the tr-RKerr and tr-SHG responses. Based on the previous theory, we can write the complex electromagnon coordinate as

$$\tilde{Q}(t) = (Q' + iQ'')e^{i\omega t}, \tag{57}$$

where Q' and Q'' correspond to the time-symmetric (*i.e.*, polarization) and time-antisymmetric (*i.e.*, magnetization) components of the electromagnon modulation. We can then expand the complex Kerr rotation angle as

$$\Delta\tilde{\theta}_\text{K}(t) \propto \sum_{i,j} A_{ij} \frac{\partial\chi^{(1)}_{ij}}{\partial\tilde{Q}} \tilde{Q}(t) \tag{58}$$

$$\propto \left[A_\varepsilon \frac{\partial\varepsilon_{xy}}{\partial Q'} Q' + iA_G \frac{\partial G_z}{\partial Q''} Q'' \right] e^{i\omega t}, \tag{59}$$

where A_{ij} encodes the dielectric contributions to the Kerr rotation (primarily $\chi^{(1)}_{xy}$ and $\chi^{(1)}_{yx}$), and we have imposed the symmetry of the respective tensor elements. Similarly, the complex nonlinear polarization can be written as

$$\Delta\tilde{P}^{(2)}_i(\phi, t) \propto \sum_{j,k} a_{jk}(\phi) \frac{\partial\chi^{(2)}_{ijk}}{\partial\tilde{Q}} \tilde{Q}(t) \tag{60}$$

$$\propto \sum_{j,k} a_{jk}(\phi) \frac{\partial\chi^{(2)}_{ijk}}{\partial Q'} Q' e^{i\omega t}, \tag{61}$$

where $a_{jk}(\phi)$ encodes the anisotropy of the optical SHG signal. Since the probe absorption is negligible, $\frac{\partial \chi_{ijk}^{(2)}}{\partial \tilde{Q}}$ is necessarily real and, based upon the discussion in Supplementary Note 3, also time-reversal symmetric. We can recover our signal by taking the real parts of the Kerr rotation and the nonlinear polarization. This yields

$$\Delta\theta_K(t) \propto -A_G \frac{\partial G_z}{\partial Q''} Q'' \sin(\omega t) \tag{62}$$

$$\Delta P_i^{(2)}(\phi, t) \propto \sum_{j,k} a_{jk}(\phi) \frac{\partial \chi_{ijk}^{(2)}}{\partial Q'} Q' \cos(\omega t). \tag{63}$$

Note we have used the fact that, in our experimental geometry, $\left|A_\varepsilon \frac{\partial \varepsilon_{xy}}{\partial Q'}\right| \ll \left|A_G \frac{\partial G_z}{\partial Q''}\right|$, as shown in the previous section. Hence, the electromagnon oscillations in the tr-RKerr and tr-SHG signals exhibit a $\pi/2$ phase shift based on their independent sensitivities to the magnetization and polarization modulations, respectively.

Supplementary Note 6: Dynamical magnetoelectric coupling

Having established the independent sensitivities of tr-SHG and tr-RKerr to the transient electric polarization and magnetization, respectively, we can now estimate the dynamical magnetoelectric coupling constant by comparing the corresponding oscillatory features. We focus on the EM_o mode due to its substantial symmetry-allowed out-of-plane magnetization and examine the relative tr-SHG and tr-RKerr modulations at an incident pump fluence of 1.65 mJ/cm^2.

We begin by writing the first-order dynamical magnetoelectric response at angular frequency ω[36]:

$$\Delta P_i(\omega) = \epsilon_0 \chi^e_{ii}(\omega)\Delta E_i(\omega) + \alpha_{ij}(\omega)\Delta H_j(\omega), \tag{64a}$$

$$\Delta M_j(\omega) = \chi^m_{jj}(\omega)\Delta H_j(\omega) + \frac{1}{\mu_0}\alpha_{ij}(\omega)\Delta E_i(\omega). \tag{64b}$$

Here, α_{ij} is the dynamical magnetoelectric coupling tensor that couples the electric polarization P_i, electric field E_i, magnetizing field H_i, and magnetization M_i. Likewise, χ^e_{ii} and χ^m_{jj} are the linear electric and magnetic susceptibility tensor elements, and ϵ_0 and μ_0 are the dielectric permittivity and magnetic permeability of free space, respectively. The elements α_{ij} are responsible for a variety of optical phenomena, including directional dichroism[37,38] (by the off-diagonal elements) and natural optical activity/gyrotropic birefringence[39–41] (by the diagonal elements). In general, α_{ij} is a complex quantity even in a dissipationless material whose real and imaginary components represent the amplitude of the magnetoelectric response that is in-phase and $\pi/2$ out-of-phase, respectively. In NiI_2, the evolution of either electromagnon produces magnetization and polarization responses that are nearly parallel to one another, which will induce gyrotropic birefringence and optical activity via the diagonal elements of the magnetoelectric response via the in-phase and out-of-phase responses, respectively. Our theoretical model proves that the magnetization and polarization oscillate almost completely out-of-phase (Fig. S7, Supplementary Note 2), indicating that the optical activity is the dominant response from the electromagnons.

Here, we estimate the intrinsic coupling characterizing the electric polarization and magnetization of the electromagnons via two procedures. For the first, we consider the microscopic picture centered upon a material whose electromagnons evolve freely in the absence of a resonant external terahertz field, *i.e.*, $E(\omega) = B(\omega) = 0$. In this case, using $H_i(\omega) = -M_i(\omega)$, we write the magnetoelectric constant based upon Eq. 64a as

$$\Delta P_i(\omega) = -\alpha_{ij}(\omega)\Delta M_j(\omega). \tag{65}$$

Alternatively, one can consider the macroscopic dynamical magnetoelectric response in the terahertz regime under the assumption that $\chi^m_{jj} = 0$ and $\chi^e_{ii} \gg \frac{1}{\sqrt{\epsilon_0\mu_0}}\alpha_{ji}$, which, following Eqs. 64a and 64b, yield an electromagnon response of

$$\Delta M_j(\omega) \approx \frac{c^2}{\chi^e_{ii}}\alpha_{ij}(\omega)\Delta P_i(\omega). \tag{66}$$

As will be demonstrated shortly, both Eqs. 65 and 66 provide similar estimates of the magnetoelectric constant of the material. From the anisotropic tr-SHG signal, we estimate that the EM_o

amplitude in terms of the nonlinear optical susceptibility is $\sim$ 20 fm/V (see Supplementary Note 4). Based upon the static susceptibility of $\sim$ 20 pm/V and the reported bulk polarization of single-domain NiI_2[3], P_{el} = 9.2$\times 10^{-14}$ C/m, when converted to a unit cell of theoretical monolayer height (*i.e.*, 1/3 the standard unit cell or $c = 6.59$ Å), the observed two-dimensional polarization modulation is

$$\begin{aligned}\Delta P^x_{EM_o} &= \frac{\Delta\chi^{(2)}_{EM_o}}{\chi^{(2)}_{yyy}} P_{el} \\ &\approx 1\times 10^{-3} P_{el} \\ &\approx 9.2\times 10^{-17}\text{C/m}.\end{aligned}$$

From tr-RKerr, we obtain an approximate EM_o amplitude in terms of the Kerr rotation of $\sim$ 4 μrad at 1.65 mJ/cm^2 (see Fig. S14). The corresponding magnetization modulation can be written as

$$\Delta M^z_{EM_o} = \frac{\rho_{EM}\mu_{EM_o}}{A}, \tag{67}$$

where μ_{EM_o} is the magnetic dipole moment of EM_o ($\approx 0.7\mu_B$ from calculations, where μ_B is the Bohr magneton), ρ_{EM} is the EM_o density per unit cell, and A is the area of the unit cell. From the transfer matrix calculations detailed in Supplementary Note 5, ρ_{EM} can be obtained from the Kerr rotation via $\theta_K \approx 0.0023\rho_{EM}$. Thus, given the two-dimensional magnetic unit cell with a = 23.95 Å, b = 3.95 Å (perpendicular to each other), we obtain a two-dimensional magnetization modulation of

$$\begin{aligned}\Delta M^z_{EM_o} &\approx \frac{\Delta\theta_K}{0.0023}\frac{0.7\mu_B}{ab} \\ &\approx 1.2\times 10^{-8}\ \text{A}.\end{aligned}$$

To compare these dynamics, we first convert the measured in-plane polarization ($||x$) and out-of-plane magnetization ($||z$) response and project both onto the EM_o axis $\kappa \sim (\hat{\mathbf{x}} + \sqrt{2}\hat{\mathbf{z}})/\sqrt{3}$. Doing so while taking into account the observed $\pi/2$ out-of-phase dynamics yields a magnetoelectric constant $\alpha_{\kappa\kappa}$ at the EM_o resonance frequency ω_o of

$$\begin{aligned}|\text{Im}\{\alpha_{\kappa\kappa}(\omega_o)\}| &= \left|\frac{\Delta P^\kappa_{EM_o}}{\Delta M^\kappa_{EM_o}}\right| \\ &\approx 11\times 10^3\ \text{ps m}^{-1},\end{aligned}$$

utilizing Eq. 65. By performing the same estimation via Eq. 66 and $\chi^e(\omega_o) \sim 11$[42], we obtain

$$\begin{aligned}|\text{Im}\{\alpha_{\kappa\kappa}(\omega_o)\}| &= \left|\frac{\chi^e\Delta M^\kappa_{EM_o}}{c^2\Delta P^\kappa_{EM_o}}\right| \\ &\approx 11\times 10^3\ \text{ps m}^{-1}.\end{aligned}$$

Remarkably, both methods yield similar values. These values agree well with our theoretical estimate of 25/7$c \approx 12\times 10^3$ ps m^{-1}, obtained using Eq. 65 by combining the electric (2.5μ_B/c) and magnetic dipole (0.7μ_B) of EM_o obtained from DFT (see Supplementary Note 2). To our knowledge, this coupling strength exceeds that of all known magnetoelectric materials in

the terahertz spectral range (see Table S7), and in particular, exceeds those responsible for the natural optical activity or gyrotropic birefringence in such materials, being equivalent to a colossal polarization rotation of $\eta \approx 1000°$/mm.

Optical phenomena from magnetoelectric coupling. Several distinctive optical phenomena, including directional dichroism, natural optical activity, and gyrotropic birefringence, arise from the magnetoelectric coupling tensor. Here, we will review the differences and similarities between these processes, along with their origins from the magnetoelectric tensor. Firstly, directional dichroism refers to the nonreciprocal absorption of light traveling through a material in opposite directions. Microscopically, it originates from the off-diagonal element of the magnetoelectric tensor, $\alpha_{ij}, i \neq j$, and is strongest for light propagating along the plane normal $\hat{\mathbf{e}}_i \times \hat{\mathbf{e}}_j$. Intuitively, this form of magnetoelectric interaction can be considered as a source term for light emission with the same polarization as the incident radiation, *e.g.*, the $H_j(\omega)$ of light will induce $E_i(\omega)$. However, the axial nature of this coupling means that the induced light fields have opposite phases for opposite propagation directions. Hence, in one direction, the magnetoelectric coupling will emit light that deconstructively interferes with the incident radiation, thus reinforcing the natural absorption of light in the material. In the other direction, the induced light will constructively interfere with the incident radiation, thus counteracting that same absorption.

The other two processes, natural optical activity and gyrotropic birefringence, are both optical phenomena that induce a complex polarization rotation of light.[43] The former can be described by the reciprocal birefringence of left- and right-circularly polarized light, thus serving as an inherent probe of the material's chirality. In contrast, the latter can be described by a non-reciprocal rotation of the material's principal optical axis. Conceptually, the two can be distinguished in that the eigenpolarizations of the former are circular, while those of the latter are linear. Another major difference is that natural optical activity can occur even in systems that preserve time-reversal symmetry, while gyrotropic birefringence requires broken time-reversal symmetry and typically scales with an applied magnetic field. Microscopically, the two processes originate from the diagonal components of the magnetoelectric coupling tensor α_{ii}, which serves as a source term for light emission that is polarized orthogonal to the incident light radiation, *e.g.*, the $H_i(\omega)$ of light will induce $E_i(\omega) \parallel H_i(\omega)$. The two differ because natural optical activity and gyrotropic birefringence originate from the imaginary and real parts of α_{ii}, respectively, in a non-dissipative system. The former results in a $\pi/2$ out-of-phase emission of the orthogonally polarized light, leading to ellipticity, while the latter results in an in-phase emission, leading to rotation.

Material	$\omega/2\pi$ (THz)	H (T)	T (K)	$T_{\text{multiferroic}}$	ij	α_{ij} (ps/m)	Resonance
NiI_2 (EM_o)	0.93	0	2.4	$T < 60$ K	Im$[\kappa\kappa]^\dagger$	11000	IDM
$(Fe, Zn)_2Mo_3O_8$[40]	1.4	0	5	$T < 60$ K	Im$[xx - zz]^\dagger$	140	ES
$Eu_{0.7}Y_{0.3}MnO_3$[44]	0.55	5	4	$T < 24$ K	Im$[xx]^\dagger$	770	ES + AFMR
$Eu_{0.55}Y_{0.45}MnO_3$[44]	0.10<br>0.58	7<br>7	4	$T < 24$ K	Im$[xz]$<br>Im$[xz]$	1100<br>300	IDM<br>ES
$Ba_2CoGe_2O_7$[39]	1.0	7	5	$T < 6.5$ K	Im$[xx + yy]/2^\dagger$	500	ES
CuO[45]	0.67	0	214	212 K $< T <$ 231 K	Re$[xx - yy]/2^\dagger$	17	IDM
$Gd_{0.5}Tb_{0.5}MnO_3$[38]	0.25<br>0.67	7<br>7	8.3	$T < 25$ K	Im$[xz]$<br>Im$[xz]$	1840<br>330	IDM<br>ES
$CuFe_{0.965}Ga_{0.035}O_2$[43]	0.25	0	4	$T < 7$ K	Re$[xx - yy]/2^\dagger$	244	IDM
$LiCoPO_4$[46]	1.2	0	2	$T < 22.5$ K	Im$[xz]$	32	ES

Table S7 | Dynamical magnetoelectric coupling strengths in different materials. Dynamical $\alpha_{ij}(\omega)$ for various magnetoelectric materials measured in resonance with characteristic collective modes. Indicated resonances include electrostriction-based electromagnons (ES), inverse Dzyaloshinskii–Moriya interaction-based electromagnons (IDM), and antiferromagnetic resonances (AFMR). † In typical frequency domain measurements, dissipation in the system will exchange the real and imaginary parts of α_{ij} at resonance. Thus, observations of natural optical activity in CuO and $CuFe_{0.965}Ga_{0.035}O_2$ show a peak in Re$[\alpha_{ii}]$ which is still comparable to our time-domain observation of Im$[\alpha_{ii}]$ in NiI_2. Similarly, the gyrotropic birefringence in $(Fe, Zn)_2Mo_3O_8$, $Eu_{0.7}Y_{0.3}MnO_3$, and $Ba_2CoGe_2O_7$ is observed in Im$[\alpha_{ii}]$ and not Re$[\alpha_{ii}]$.

Supplementary Note 7: Pump polarization dependence

We performed a series of tr-SHG measurements while varying the incident linear pump polarization and helicity. We then fitted the oscillations of the EM_o mode to a decaying sinusoid of the form

$$Ae^{-\gamma t}\cos(\omega t + \Phi), \tag{68}$$

where A is the amplitude, γ is the decay constant, ω is the angular frequency, and Φ is phase of the oscillator. The resulting amplitudes and initial phase of the oscillations are shown in Fig. S16. Notably, we observed that the electromagnon oscillations show little dependence on the linear polarization of the incident pump, which is inconsistent with coherent excitation via the inverse Cotton-Mouton effect[47,48]. The small residual dependence of the amplitude (<10%) may be attributed to the weak birefringence in the material. Similarly, a comparison of the EM_o signal as a function of the helicity of the light reveals little dependence, which also excludes the inverse Faraday effect behind coherent magnon generation[49,50]. Combining these observations, we believe that the likely excitation mechanism is mediated by the photoexcited carrier density produced by the residual absorption of the 1.13 eV pump pulse. Recent studies have identified possible microscopic mechanisms that can lead to such results in various canted and zig-zag antiferromagnets[51–53]. Possible extensions of these theories to the case of spiral magnetic orders that lead to type-II multiferroicity may shed light on the physics at play in NiI_2.

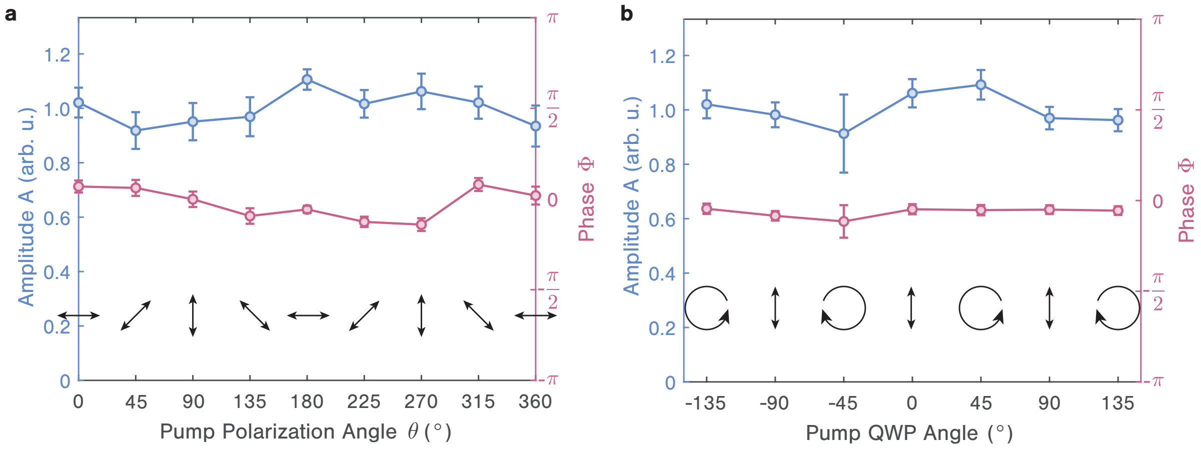

Fig. S16 | Pump polarization dependent tr-SHG signal. a, The photoexcited EM_o amplitude A (*blue*) and initial phase Φ (*red*) as a function of the linear pump polarization angle, θ, where $\theta = 90°$ corresponds to excitation parallel to the C_2 axis. **b,** Similar EM_o amplitude and initial phase as a function of pump helicity. The measurements were performed with an incident pump fluence of 1.65 mJ/cm^2 at 2.4 K. QWP: quarter-wave plate. All error bars show the 95% confidence intervals obtained from the fits.

Supplementary Figures

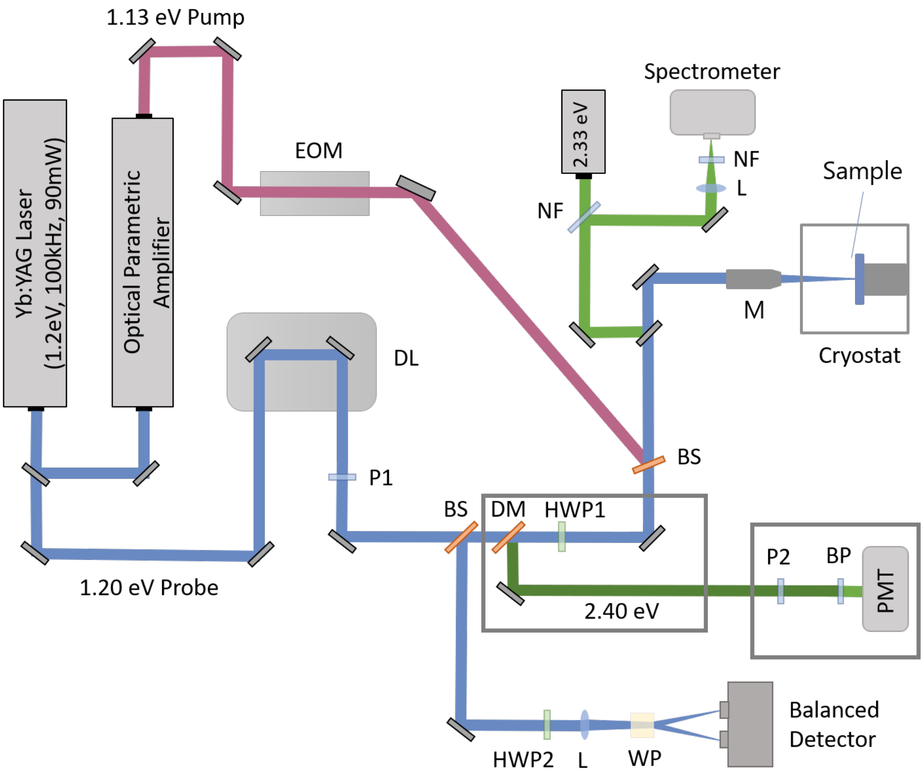

Fig. S17 | Schematic diagram of the experimental setup. The samples could be imaged and measured using different techniques, including spontaneous Raman scattering, tr-SHG, and tr-RKerr, under the same experimental conditions. The red and blue lines indicate the pump and probe paths in tr-SHG and tr-RKerr, respectively. The light green, green, and split blue lines represent the detection of the Raman, tr-SHG, and tr-RKerr signals, respectively. DL: delay stage; P: polarizer; BS: beamsplitter; DM: dichroic mirror; HWP: half-wave plate; M: microscope objective; BP: bandpass filter; L: lens; WP: Wollaston prism; NF: notch filter.

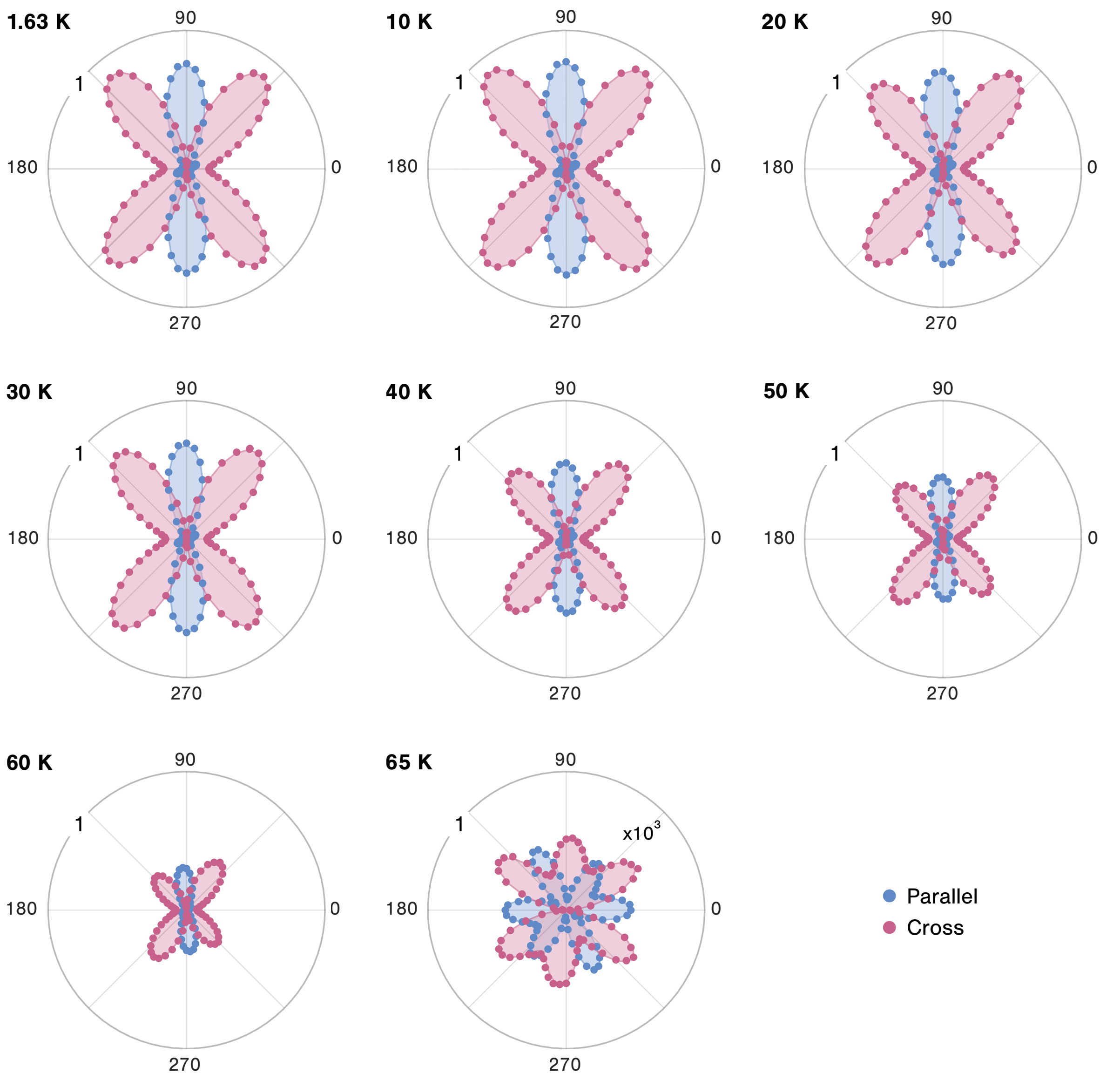

Fig. S18 | Temperature-dependent static SHG polarimetry patterns. SHG polarimetry patterns measured in perpendicular (*red*) and parallel (*blue*) polarization configurations at different temperatures. Note that the pattern observed at 65 K has been scaled by 10^3 for clarity. Above $T_{HM} \simeq 60$ K, the SHG signal shows a six-fold anisotropy. When the temperature drops below T_{HM}, a strong SHG emission with a two-fold anisotropy pattern appears, indicating the emergence of an electric polarization and the reduction in crystal symmetry ($\bar{3}m \to 2$).

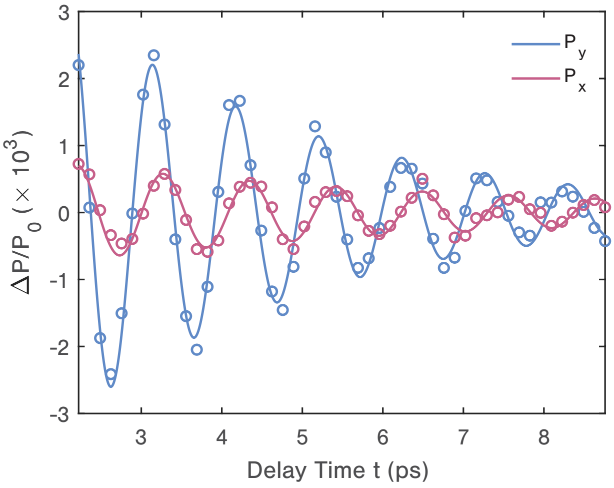

Fig. S19 | Electromagnon polarization evolution. Evolution of the electric polarization due to coherent electromagnon oscillations shown in Fig. 3c of the main text is plotted as a function of pump-probe delay. Fits to a damped oscillator (*solid lines*) are shown alongside the experimental data (*circles*).

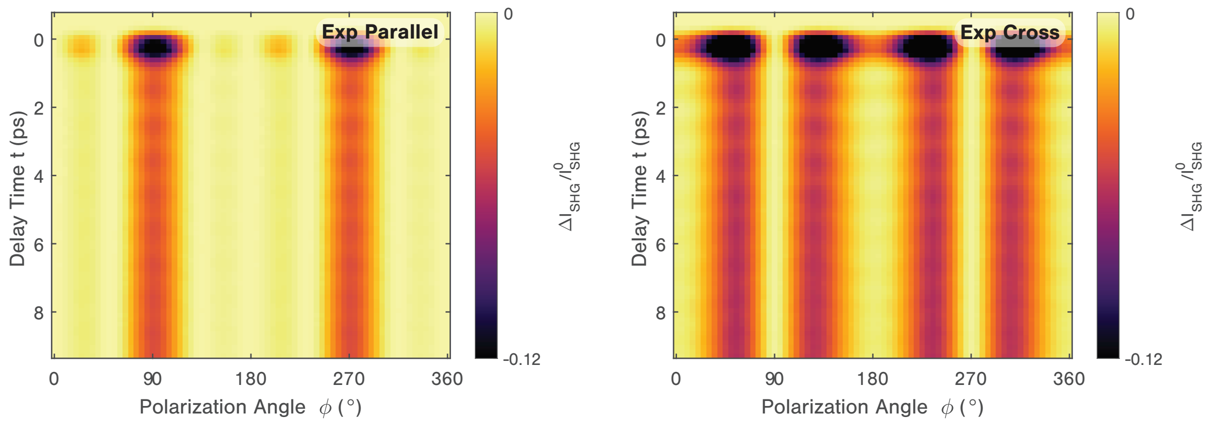

Fig. S20 | Raw tr-SHG polarimetry signals. Raw anisotropic tr-SHG signals collected in both parallel (*left*) and crossed (*right*) polarized configurations. Data corresponding to the same signals are shown in Fig. 2d,f of the main text, but without the incoherent background. Signals are normalized to the maximum static SHG signal of $\sim 500\ \mu$V.

References

1. Siegler, T. D. *et al.* Deliquescent chromism of nickel(II) iodide thin films. *Langmuir* **35**, 2146–2152 (2019).

2. Song, Q. *et al.* Evidence for a single-layer van der Waals multiferroic. *Nature* **602**, 601–605 (2022).

3. Kurumaji, T. *et al.* Magnetoelectric responses induced by domain rearrangement and spin structural change in triangular-lattice helimagnets NiI_2 and CoI_2. *Phys. Rev. B* **87**, 014429 (2013).

4. Riedl, K. *et al.* Microscopic origin of magnetism in monolayer 3D transition metal dihalides. *Phys. Rev. B* **106**, 035156 (2022).

5. Maksimov, P. A., Zhu, Z., White, S. R. & Chernyshev, A. L. Anisotropic-exchange magnets on a triangular lattice: Spin waves, accidental degeneracies, and dual spin liquids. *Phys. Rev. X* **9**, 021017 (2019).

6. Katsura, H., Nagaosa, N. & Balatsky, A. V. Spin current and magnetoelectric effect in noncollinear magnets. *Phys. Rev. Lett.* **95**, 057205 (2005).

7. Wu, S. *et al.* Layer thickness crossover of type-II multiferroic magnetism in NiI_2. *arXiv:2307.10686* (2023).

8. Oleksy, C. Critical phenomena in systems with bilinearly coupled order parameters—application: magnetic structures of uranium compounds U_3X_4. *Acta Phys. Pol. A* **66**, 665–676 (1984).

9. Zhang, B., Hou, Y., Wang, Z. & Wu, R. First-principles studies of spin-phonon coupling in monolayer $Cr_2Ge_2Te_6$. *Phys. Rev. B* **100**, 224427 (2019).

10. Fumega, A. O. & Lado, J. L. Microscopic origin of multiferroic order in monolayer NiI_2. *2D Mater.* **9**, 025010 (2022).

11. King-Smith, R. D. & Vanderbilt, D. Theory of polarization of crystalline solids. *Phys. Rev. B* **47**, 1651–1654 (1993).

12. Xiang, H., Kan, E., Zhang, Y., Whangbo, M.-H. & Gong, X. General theory for the ferroelectric polarization induced by spin-spiral order. *Phys. Rev. Lett.* **107**, 157202 (2011).

13. Li, X. *et al.* Realistic spin model for multiferroic NiI_2. *Phys. Rev. Lett.* **131**, 036701 (2023).

14. Xiang, H., Kan, E., Wei, S.-H., Whangbo, M.-H. & Gong, X. Predicting the spin-lattice order of frustrated systems from first principles. *Phys. Rev. B* **84**, 224429 (2011).

15. Xiang, H., Lee, C., Koo, H.-J., Gong, X. & Whangbo, M.-H. Magnetic properties and energy-mapping analysis. *Dalton Trans.* **42**, 823–853 (2013).

16. Šabani, D., Bacaksiz, C. & Milošević, M. V. Ab initio methodology for magnetic exchange parameters: Generic four-state energy mapping onto a Heisenberg spin Hamiltonian. *Phys. Rev. B* **102**, 014457 (2020).

17. Ju, H. *et al.* Possible persistence of multiferroic order down to bilayer limit of van der Waals material NiI_2. *Nano Lett.* **21**, 5126–5132 (2021).

18. Jiang, Y. *et al.* Dilemma in optical identification of single-layer multiferroics. *Nature* **619**, E40–E43 (2023).

19. Song, Q. *et al.* Reply to: Dilemma in optical identification of single-layer multiferroics. *Nature* **619**, E44–E46 (2023).

20. Fiebig, M., Pavlov, V. V. & Pisarev, R. V. Second-harmonic generation as a tool for studying electronic and magnetic structures of crystals: review. *J. Opt. Soc. Am. B* **22**, 96–118 (2005).

21. Fiebig, M., Fröhlich, D., Krichevtsov, B. B. & Pisarev, R. V. Second harmonic generation and magnetic-dipole-electric-dipole interference in antiferromagnetic Cr_2O_3. *Phys. Rev. Lett.* **73**, 2127–2130 (1994).

22. Xiao, R.-C. *et al.* Classification of second harmonic generation effect in magnetically ordered materials. *npj Quantum Mater.* **8**, 62 (2023).

23. Wagnière, G. H. & Woźniak, S. Nonlinear Optical Properties. In Lindon, J. C., Tranter, G. E. & Koppenaal, D. W. (eds.) *Encyclopedia of Spectroscopy and Spectrometry (Third Edition)*, 375–387 (Academic Press, Oxford, 2017).

24. Sun, Z. *et al.* Giant nonreciprocal second-harmonic generation from antiferromagnetic bilayer CrI_3. *Nature* **572**, 497–501 (2019).

25. Song, W., Fei, R., Zhu, L. & Yang, L. Nonreciprocal second-harmonic generation in few-layer chromium triiodide. *Phys. Rev. B* **102**, 045411 (2020).

26. Xiao, R.-C. *et al.* Comprehensive classification of second harmonic generation effect in magnetic materials. *arXiv:2211.04354* (2022).

27. Meier, D. *et al.* Second harmonic generation on incommensurate structures: The case of multiferroic $MnWO_4$. *Phys. Rev. B* **82**, 155112 (2010).

28. Lottermoser, T., Meier, D., Pisarev, R. V. & Fiebig, M. Giant coupling of second-harmonic generation to a multiferroic polarization. *Phys. Rev. B* **80**, 100101 (2009).

29. Fiebig, M., Lottermoser, T. & Pisarev, R. V. Spin-rotation phenomena and magnetic phase diagrams of hexagonal $RMnO_3$. *J. Appl. Phys.* **93**, 8194–8196 (2003).

30. Fröhlich, D., Leute, S., Pavlov, V. V. & Pisarev, R. V. Nonlinear optical spectroscopy of the two-order-parameter compound $YMnO_3$. *Phys. Rev. Lett.* **81**, 3239–3242 (1998).

31. Yee, K. J., Lee, K. G., Oh, E., Kim, D. S. & Lim, Y. S. Coherent optical phonon oscillations in bulk GaN excited by far below the band gap photons. *Phys. Rev. Lett.* **88**, 105501 (2002).

32. Wu, A. Q. & Xu, X. Coherent phonon excitation in bismuth. *Appl. Surf. Sci.* **253**, 6301–6304 (2007).

33. Hase, M. & Kitajima, M. Interaction of coherent phonons with defects and elementary excitations. *J. Phys. Condens. Matter* **22**, 073201 (2010).

34. Bossini, D. *et al.* Macrospin dynamics in antiferromagnets triggered by sub-20 femtosecond injection of nanomagnons. *Nat. Commun.* **7**, 10645 (2016).

35. De, A. *et al.* Coherent and incoherent magnons induced by strong ultrafast demagnetization in thin permalloy films. *Phys. Rev. B* **109**, 024422 (2024).

36. Fiebig, M. Revival of the magnetoelectric effect. *J. Phys. D Appl. Phys.* **38**, R123 (2005).

37. Takahashi, Y., Shimano, R., Kaneko, Y., Murakawa, H. & Tokura, Y. Magnetoelectric resonance with electromagnons in a perovskite helimagnet. *Nat. Phys.* **8**, 121–125 (2012).

38. Takahashi, Y., Yamasaki, Y. & Tokura, Y. Terahertz magnetoelectric resonance enhanced by mutual coupling of electromagnons. *Phys. Rev. Lett.* **111**, 037204 (2013).

39. Bordács, S. *et al.* Chirality of matter shows up via spin excitations. *Nat. Phys.* **8**, 734–738 (2012).

40. Kurumaji, T. *et al.* Optical magnetoelectric resonance in a polar magnet $(Fe, Zn)_2Mo_3O_8$ with axion-type coupling. *Phys. Rev. Lett.* **119**, 077206 (2017).

41. Kuzmenko, A. M. *et al.* Sign change of polarization rotation under time or space inversion in magnetoelectric $YbAl_3(BO_3)_4$. *Phys. Rev. B* **99**, 224417 (2019).

42. Kim, J. H. *et al.* Terahertz evidence of electromagnon excitations in the multiferroic van der Waals insulator NiI_2. *Phys. Rev. B* **108**, 064414 (2023).

43. Iguchi, S., Masuda, R., Seki, S., Tokura, Y. & Takahashi, Y. Enhanced gyrotropic birefringence and natural optical activity on electromagnon resonance in a helimagnet. *Nat. Commun.* **12**, 6674 (2021).

44. Ogino, M., Kaneko, Y., Tokura, Y. & Takahashi, Y. Optical magnetoelectric effects resonantly enhanced via electromagnons in cycloidal helimagnets $Eu_{1-x}Y_xMnO_3$. *Phys. Rev. B* **108**, 024418 (2023).

45. Masuda, R., Kaneko, Y., Tokura, Y. & Takahashi, Y. Electric field control of natural optical activity in a multiferroic helimagnet. *Science* **372**, 496–500 (2021).

46. Kocsis, V. *et al.* Identification of antiferromagnetic domains via the optical magnetoelectric effect. *Phys. Rev. Lett.* **121**, 057601 (2018).

47. Kalashnikova, A. *et al.* Impulsive generation of coherent magnons by linearly polarized light in the easy-plane antiferromagnet $FeBO_3$. *Phys. Rev. Lett.* **99**, 167205 (2007).

48. Afanasiev, D. *et al.* Controlling the anisotropy of a van der Waals antiferromagnet with light. *Sci. Adv.* **7**, eabf3096 (2021).

49. Kirilyuk, A., Kimel, A. V. & Rasing, T. Ultrafast optical manipulation of magnetic order. *Rev. Mod. Phys.* **82**, 2731 (2010).

50. Toyoda, S., Kruppe, J., Yamakawa, K., Analytis, J. & Orenstein, J. Phase control of spin waves in the van der Waals antiferromagnet $NiPS_3$. *Phys. Rev. B* **109**, 064408 (2024).

51. Mikhaylovskiy, R. V. *et al.* Ultrafast optical modification of exchange interactions in iron oxides. *Nat. Commun.* **6**, 8190 (2015).

52. Belvin, C. A. *et al.* Exciton-driven antiferromagnetic metal in a correlated van der Waals insulator. *Nat. Commun.* **12**, 4837 (2021).

53. Allington, C. J. *et al.* Distinct optical excitation mechanisms of a coherent magnon in a van der Waals antiferromagnet. *arXiv:2402.17041* (2024).
